# In vivo mitochondrial oxygen consumption during LPS-induced endotoxemia: a controlled experimental study in swine

**DOI:** 10.1186/s40635-026-00951-z

**Published:** 2026-07-20

**Authors:** Calvin Jay de Wijs, Jan van den Brink, Patricia A.C. Specht, Mariëlle van der Kaaij, Harold N.J. Raat, Bülent Ergin, Robert Jan Stolker, Egbert G. Mik, Floor A. Harms

**Affiliations:** 1 Laboratory of Experimental Anesthesiology, Department of Anesthesiology, Erasmus MC, PO Box 2040, 3000 CA , Rotterdam, the Netherlands; 2 Laboratory of Translational Intensive Care, Department of Intensive Care Adult, Erasmus, Rotterdam, MC the Netherlands

**Keywords:** Swine, Endotoxemia, Mitochondria, Microcirculation, Renal injury, Lactate

## Abstract

**Background:**

Sepsis and septic shock are life-threatening syndromes characterized by complex systemic effects, including alterations in tissue oxygenation, mitochondrial function, and organ performance. Despite advances in supportive care, management remains challenging and is largely focused on maintaining adequate perfusion through fluids and vasoactive agents. To better characterize early systemic responses to endotoxemia under different hemodynamic conditions, this study used a swine lipopolysaccharide (LPS)-induced endotoxemia model with graded hypotension exposure. Animals were allocated to a control group (MAP maintained > 80 mmHg) and two LPS-groups defined by different MAP thresholds for initiating hemodynamic support (LPS-1: <80 mmHg; LPS-2: <65 mmHg), with a focus on early mitochondrial and microcirculatory (dys)function.

**Results:**

LPS administration induced marked systemic responses, including tachycardia, metabolic stress, and organ dysfunction. Microcirculatory alterations were present but quantitatively modest. In vivo mitochondrial oxygen tension (mitoPO_2_) remained largely preserved or was only transiently affected, whereas mitochondrial oxygen consumption (mitoVO_2_) was reduced or showed a blunted time course in the epidermis and liver. In contrast, ex vivo analyses revealed increased mitochondrial respiratory flux in peripheral blood mononuclear cells during early endotoxemia. More permissive hypotension was associated with a greater cumulative hypotension burden and stronger renal injury signals, including higher neutrophil gelatinase-associated lipocalin concentrations and reduced renal clearance. Arterial lactate concentrations increased during endotoxemia and were inversely associated with hepatic indocyanine green plasma disappearance rate.

**Conclusion:**

Early LPS-induced endotoxemia was associated with reduced or blunted mitoVO_2_ despite largely preserved mitoPO_2_, consistent with partial uncoupling between mitochondrial oxygen availability and utilization. Microcirculatory alterations were modest and unlikely to fully account for the observed changes in mitoVO_2_. Supportive ex vivo and organ function findings indicated increased mitochondrial respiratory activity and early renal vulnerability under more permissive hypotension. Together these findings highlight that mitoVO_2_ is a valuable parameter in combination with mitoPO_2_ to probe tissue-level mitochondrial function during early endotoxemia.

**Supplementary Information:**

The online version contains supplementary material available at 10.1186/s40635-026-00951-z.

## Background

Sepsis and septic shock are life-threatening syndromes characterized by widespread physiological, pathological, and biochemical abnormalities triggered by infection [1]. These conditions are major global health concerns due to their high mortality and morbidity rates. Systemic effects of sepsis include tissue ischemia, cytopathic injury, apoptosis, mitochondrial dysfunction, microcirculatory alterations, and multiple organ failure [2, 3]. Recent research highlights the complex interplay between inflammatory pathways, hemodynamic changes, and mitochondrial dysfunction in sepsis, complicating both diagnosis and treatment [4–7].

Given the multifactorial nature of sepsis, treatment focuses on maintaining adequate tissue perfusion through early administration of intravenous fluids and vasoactive agents [8]. However, while fluid administration may transiently modulate inflammation and improve microvascular perfusion, excessive fluid accumulation (fluid overload) is associated with adverse outcomes and does not necessarily translate into improved clinical outcomes [9]. When fluid resuscitation alone is insufficient, vasoactive agents should be initiated to maintain a mean arterial pressure (MAP) of 65 mmHg, as higher MAP targets have not demonstrated additional benefit [8]. For instance, Asfar et al. found that targeting an MAP of 80–85 mmHg required significantly more vasopressor use yet failed to improve outcomes compared to a target of 65–70 mmHg [10]. Furthermore, a MAP below 60 mmHg in humans is associated with decreased organ perfusion [11]. These findings suggest that conventional hemodynamic management, focusing mainly on global parameters such as blood pressure and cardiac output, may be inadequate in sepsis.

Emerging evidence suggests that microcirculatory parameters can provide critical insights into the adequacy of tissue perfusion and oxygenation, which are essential for optimizing patient outcomes [12, 13]. However, the clinical adoption of microcirculation-based hemodynamic management has been limited by technical challenges, difficulties in data interpretation, and the lack of standardized guidelines [12, 14]. An additional promising approach is the monitoring of mitochondrial oxygenation and consumption. Our group previously demonstrated the feasibility of assessing mitochondrial oxygen consumption (mitoVO_2_) in a rat model [15]. This technique is now clinically applicable using the CE-marked COMET^®^ monitor (Photonics Healthcare B.V., Utrecht, the Netherlands), enabling real-time mitochondrial function assessment at the bedside [16, 17]. Before conducting measurements in the complex and heterogeneous population of septic patients [18], we aimed to better understand the systemic effects of sepsis and septic shock in an animal model.

In this study, we aimed to investigate mitochondrial function and oxygen utilization during sepsis, with a particular focus on in vivo mitochondrial oxygen consumption (mitoVO_2_). Our primary objective was to characterize the dynamics of mitoVO_2_ during and after sepsis induction, building on previous findings from our rat study [15]. To enable this, we utilized a swine lipopolysaccharide (LPS) endotoxemia model, which offers important advantages over rodent models, including greater similarity to human cardiovascular physiology, the capacity for clinically relevant hemodynamic and microcirculatory monitoring, and the ability to perform repeated serial measurements using standard clinical instrumentation [19, 20].

In addition to mitoVO_2_, we conducted in vivo evaluations of mitochondrial oxygen tension (mitoPO_2_) and sublingual microcirculation, alongside standard hemodynamic monitoring. Ex vivo analyses included high-resolution respirometry, assessments of mitochondrial and nuclear DNA (mtDNA and nDNA), and evaluations of organ function. Together, these complementary measurements were used to contextualize mitochondrial responses within the systemic inflammatory and hemodynamic changes of sepsis. The experimental design included a control group and two LPS-exposed groups defined by differing mean arterial pressure (MAP) thresholds for initiating hemodynamic resuscitation: LPS-1 in which hemodynamic support was initiated at MAP < 80 mmHg, and LPS-2, in which hemodynamic support was initiated at MAP < 65 mmHg. These thresholds determined the timing of intervention and were intended to generate graded cumulative hypotension exposure during early endotoxemia. Accordingly, LPS-2 allowed for more permissive hypotension, enabling assessment of its impact on organ and mitochondrial function.

## Methods

### Animals

This study included 30 specific pathogen free (SPF) female Yorkshire x Norwegian Landrace swine aged 3–4 months and weighing 26–34 kg. All procedures complied with national guidelines for the care of laboratory animals and were approved by the Erasmus Medical Center (Erasmus MC), Animal Care Committee, Rotterdam, The Netherlands (DEC2115658). This article also adheres to the ARRIVE guidelines for reporting animal research.

Animals were allocated in a non-randomized, fixed alternating sequence to three groups: a control group with a MAP target > 80 mmHg (Control), LPS-1 with LPS-induced endotoxemia and initiation of hemodynamic support at MAP < 80 mmHg, and LPS-2 with initiation of support at MAP < 65 mmHg. Allocation followed the sequence Control → LPS-1 → LPS-2 until 30 animals were included to ensure balanced group distribution during the study period. Animals that died prematurely were replaced to maintain the predefined group size.

### Experimental preparations

After an overnight fast with free access to water, animals were sedated intramuscularly with tiletamine/zolazepam (6/6 mg•kg^− 1^; Virbac Laboratories, Carros, France), xylazine (2 mg•kg^− 1^; Dechra Pharmaceuticals PLC, Bladel, The Netherlands) and atropine sulfate (0.5 mg/animal; Centrafarm Services BV, Etten-Leur, The Netherlands). After 10 min, anesthesia was induced with tiletamine/zolazepam (50–100 mg/animal) and ketamine (100–300 mg/animal; Dechra Pharmaceuticals PLC, Bladel, The Netherlands) via an auricular vein cannula. Tracheal intubation was performed with a size 7.0 endotracheal tube. Anesthesia was maintained with continuous infusions of ketamine (5 mg•kg^− 1^•h^− 1^), midazolam (1.5 mg•kg^− 1^•h^− 1^; Aurobindo Pharma BV, Baarn, The Netherlands), and sufentanil (4 µg•kg^− 1^•h^− 1^; Hameln Pharma GmbH, Hameln, Germany). Muscle relaxation was achieved with rocuronium bromide (4 mg•kg^− 1^•h^− 1^; Fresenius Kabi Nederland BV, Huis ter Heide, The Netherlands).

After induction, all animals received a 500 ml bolus of colloid solution (Voluven^®^; Fresenius Kabi Nederland BV). The infusion rates of crystalloids (Sterofundin^®^ ISO 3–30 mL•kg^− 1^•h^− 1^; B. Braun, Melsungen, Germany; sodium chloride 0.9%, 2–40 mL•kg^− 1^•h^− 1^; Baxter BV, Utrecht, The Netherlands) and noradrenaline (0.01–1.8 µg•kg^− 1^•min^− 1^; Centrafarm Services BV, Breda, The Netherlands) were adjusted according to the predefined support thresholds. Magnesium sulfate (1000 mg; Eureco-Pharma BV, Ridderkerk, The Netherlands) was administered as arrhythmia prophylaxis and cefazolin (1000 mg/animal; Mylan BV, Amstelveen, The Netherlands) was given, at induction and after 4 h for infection prevention. No additional vasoactive, inotropic, or immunomodulatory agents were used.

Animals were ventilated using pressure control, with adjustments to maintain normocapnia and arterial oxygen tension (PaO_2_) within normal ranges. Normothermia (38–41 °C) was maintained using nasal temperature monitoring, heating pads, and an insulating blanket. Continuous cardiac output (CCO) was monitored via a 4 Fr thermodilution catheter in the right femoral artery, with a 3.8 Fr catheter in the left femoral artery for arterial blood pressure (ABP), heart rate (HR) monitoring, and arterial blood gas sampling. Venous access included a 7 Fr, 3-lumen catheter in the right jugular vein for blood sampling, central venous pressure (CVP) measurements, and drug/fluid administration, and a 9.5 Fr, 5-lumen catheter in the left femoral vein for additional fluid/drug administration. All catheters were placed using the Seldinger technique. Moreover, monitoring also included continuous electrocardiogram (ECG) and oxygen saturation (SpO_2_).

Immediately after central venous access was established, 2.5 mg of 5-aminolevulinic acid (ALA) (Carl Roth GmbH & Co. KG, Karlsruhe, Germany) was administered intravenously. Additionally, a 20% ALA cream was applied to the skin between the scapulae. ALA is required for optical mitoPO_2_ and mitoVO_2_ measurements, as explained below in Sect.  "MitoPO_2_ and mitoVO_2_"

In supine position, a lower midline abdominal incision was made to insert a cystostomy tube into the urinary bladder with purse-string sutures for urine collection. The pig was subsequently positioned laterally and remained in this position for the duration of the experiment. The right kidney was exposed via a flank incision, and the ureter was isolated, ligated, and cannulated with a custom-made polyethylene catheter for urine sampling. Two abdominal incisions were made access to the liver and small intestine for mitoPO_2_ and mitoVO_2_ measurements.

### Experimental design

Baseline measurements started 3 h after ALA administration. (see Sect.  "MitoPO_2_ and mitoVO_2_" for rationale). Each measurement session included mitoPO_2_ and mitoVO_2_ measurements on the kidney, liver, and intestine, as well as sublingual microcirculation assessments and the collection of arterial and venous blood samples, urine samples, and organ function measurements.

MAP was maintained > 80 mmHg during baseline in all groups. In the control group this target was maintained throughout the experiment. In LPS groups hemodynamic support was initiated once MAP decreased below the group-specific thresholds (80 mmHg in LPS-1 and 65 mmHg in LPS-2). These thresholds were selected pragmatically to induce graded hypotension exposure, as baseline MAP in swine (86–123 mmHg) is higher than in humans. Blood glucose was maintained within the normal range (3.6–6.8 mmol•L^− 1^) in all groups [21], using intravenous glucose 50% when required (B. Braun, Melsungen, Germany).

Importantly, both LPS groups were managed according to the same resuscitation principles, using fluids and noradrenaline. The predefined MAP thresholds were used to modulate the timing and intensity of hemodynamic support, thereby generating degrees of cumulative hypotension exposure during early endotoxemia, rather than to model distinct clinical resuscitation strategies.

After baseline measurements, lipopolysaccharide (5 mg•mL^− 1^, Escherichia coli O127, Sigma-Aldrich, St. Gallen SG, Switzerland) was administered intravenously in both LPS groups at an initial rate of 1.75 µg•kg⁻¹•h⁻¹, increased by 0.25 µg•kg⁻¹•h⁻¹ every 45 min until reaching 2.25 µg•kg⁻¹•h⁻¹. The dosing regimen was determined in a pilot study.

**Fig. 1:**
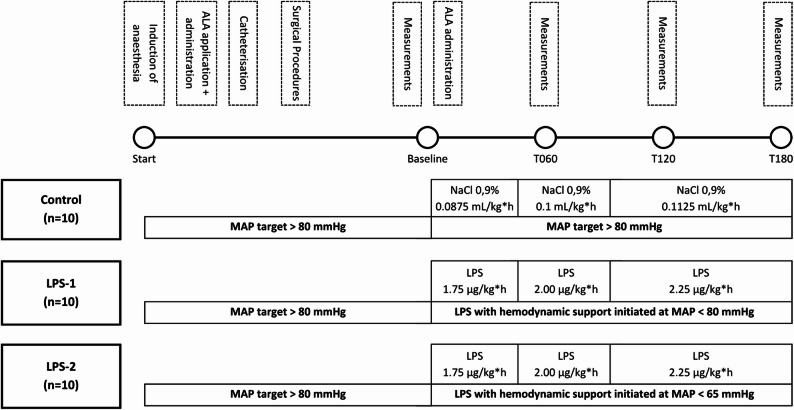
Overall experimental design

Measurements were repeated at 60, 120, and 180 min. At the end of the experiment animals were euthanized with potassium chloride overdose (Fresenius Kabi AG, Bad Homburg, Germany). The overall experimental design is shown in Fig. 1.

### In vivo measurements

#### MitoPO_2_ and mitoVO_2_

mitoPO_2_ and mitoVO_2_ were measured on the skin, renal cortex, liver, and intestinal serosa, requiring the administration of ALA (further details in Additional file 1). ALA enhanced protoporphyrin-IX (PpIX) concentration, the final precursor in the mitochondrial heme biosynthetic pathway. The PpIX-triplet state lifetime technique (PpIX-TSLT) was employed, with background and principles detailed elsewhere [16, 22, 23]. Briefly, PpIX’s triplet state reacts with oxygen, making its lifetime oxygen dependent. Upon photoexcitation, PpIX emits red delayed fluorescence, and the fluorescence lifetime is related to mitoPO_2_ via the Stern-Volmer equation. mitoPO_2_ was calculated using the rectangular distribution method (RDM) (described in Additional file 1). RDM is used to provide a reliable estimation of the average mitochondrial oxygen tension (i.e. mitoPO_2_) when oxygen concentrations are heterogeneously distributed, and the delayed fluorescence signal is multi-exponential instead of mono-exponential. The fitting function uses a simple rectangular distribution with a mean Q_m_ and a width 2δ, meaning mitochondrial PO_2_ ranges from Q_m_ – δ to Q_m_ + δ. Therefore, mitoPO_2_ as measured with the COMET is an average PO_2_ value in the mitochondria of the cells within the measuring volume. Cells close to oxygen supplying blood vessels see a higher oxygen level, while cells farther away have lower oxygen tension due to the oxygen gradient.

mitoVO_2_ is based on the oxygen disappearance rate (ODR) principle [24–28] and its measurement requires a sequence of mitoPO_2_ measurements at 1 Hz. During this sequence, mechanical pressure is applied to the measuring probe to induce stop-flow conditions, causing a time-dependent reduction in mitoPO_2_. The rate of mitoPO_2_ change during stop-flow conditions is determined from the linear portion of the curve following tissue compression [24] (further details in Additional file 1).

#### Sublingual microcirculation

A hand-held in vivo microscope, CytoCam™ (Braedius Scientific, Huizen, The Netherlands), utilizing incident dark-field (IDF) imaging, was placed on the sublingual surface. At each time point, a 100-second video clip was recorded. The device was manually held in position during measurements without the use of a mechanical holder. Efforts were made to record subsequent measurements from the same sublingual region, although exact repositioning of the imaging field could not be guaranteed. Video acquisition was not performed in a blinded manner due to the integrated nature of the experimental workflow. All clips were anonymized and analyzed in a blinded manner, with group and time point concealed. Only vessels with a diameter < 20 μm were considered. Microcirculatory parameters, including total vessel density (TVD), functional capillary density (FCD), proportion of perfused vessels (PPV), red blood cell velocity (RBCv), and tissue red blood cell perfusion (tRBCp), were analyzed using MicroTools automated software [29].

### Ex vivo mitochondrial measurements

Ex vivo mitochondrial function and circulating mitochondrial and nuclear DNA markers were assessed as supportive measurements. Detailed methodologies and protocols are provided in Additional file 2. Briefly, peripheral blood mononuclear cells (PBMCs) were isolated from arterial blood samples and analyzed using high-resolution respirometry (Oxygraph O2k, Oroboros Instruments, Innsbruck, Austria) to determine routine, leak, and maximal mitochondrial respiration under standardized conditions. In parallel, circulating mitochondrial DNA (mtDNA) and nuclear DNA (nDNA) were quantified in plasma and PBMCs using qPCR-based methods targeting mtND1 and GCG.

Histopathological assessment was performed on kidney, liver, and intestinal tissue samples obtained at the end of the experiment. Sections were processed and stained for HIF-1α expression using established immunohistochemical techniques.

### Organ function

Organ function was evaluated as a secondary outcome. Detailed measurement protocols are provided in Additional file 3. Liver function was assessed using indocyanine green plasma disappearance rate (ICG-PDR). Renal function was evaluated by creatinine clearance of the right kidney. In addition, biomarkers of organ injury were measured, including urinary neutrophil gelatinase-associated lipocalin (NGAL) and plasma intestinal fatty acid binding protein (I-FABP).

### Data analysis

#### Sample size

A power analysis was conducted based on prior findings demonstrating a significant mitoVO_2_ reduction in septic rats (5.7 ± 0.5 mmHg/s to 1.8 ± 0.3 mmHg/s post-LPS) [24]. Using these values, a two-sided power calculation (α = 0.05, power = 80%) determined that a minimum of 10 subjects per group was required to detect a significant difference. Accordingly, the study was designed to include three experimental groups, resulting in a total sample size of 30 swine.

#### Data processing

Time-weighted averages of MAP (TWA-MAP) below the thresholds of 60 and 80 mmHg were calculated by dividing the area under these thresholds within a measurement interval by the total duration of that interval. An example is provided in Additional file 4.

#### Statistical analysis

The normality of the data was assessed through visual inspection of histograms and Q-Q plots, supplemented by the Shapiro-Wilk test. Data not following a normal distribution were presented as the median and interquartile range [IQR], while normally distributed data were described as the mean ± standard deviation (SD). A threshold of *p* < 0.05 was considered statistically significant.

For graphical representation, the mean of three measurements per organ at each time point was calculated. However, in the linear mixed model (LMM), these measurements were treated as correlated observations rather than independent, to account for the interrelatedness of the data points.

LMMs were used to assess the effects of group, time point, and their interaction on various parameters, with each parameter analyzed in a separate model. This approach models all time points simultaneously and inherently accounts for baseline differences. Random effects for individual subjects were included to account for intra-subject variability and dependency. Model diagnostics were performed by assessing residual normality using Q-Q plots and checking homoscedasticity and linearity through residual versus fitted value plots. Statistical analysis was conducted using R software version 4.3.1 (R Foundation for Statistical Computing, Vienna, Austria) and packages lme4 for modeling, and lmerTest for inference of the mixed models [30].

All statistical inference regarding time effects and group-by-time interactions was based on linear mixed-effects models, with corresponding model outputs provided in Additional file 5. Descriptive statistics (median [IQR]) and figures are presented for visualization purposes. Effect sizes are reported as fixed effect estimates with full model estimates are provided in Additional file 5.

## Results

A total of 30 swine were included in the analysis, with 10 swine in each group: control, LPS-1, and LPS-2. Four swine were replaced: two in the LPS-2 group, one in the LPS-1 group, and one in the control group. The control swine was replaced due to repeated dislocation of the renal catheter, which led to a SIRS-like response, whereas the three swine replaced in the LPS groups died prematurely. Throughout the results section, tables provide descriptive median [IQR] values, while figures and reported estimates summarize the linear mixed model analyses used for statistical inference.

### Monitored parameters

**Fig. 2:**
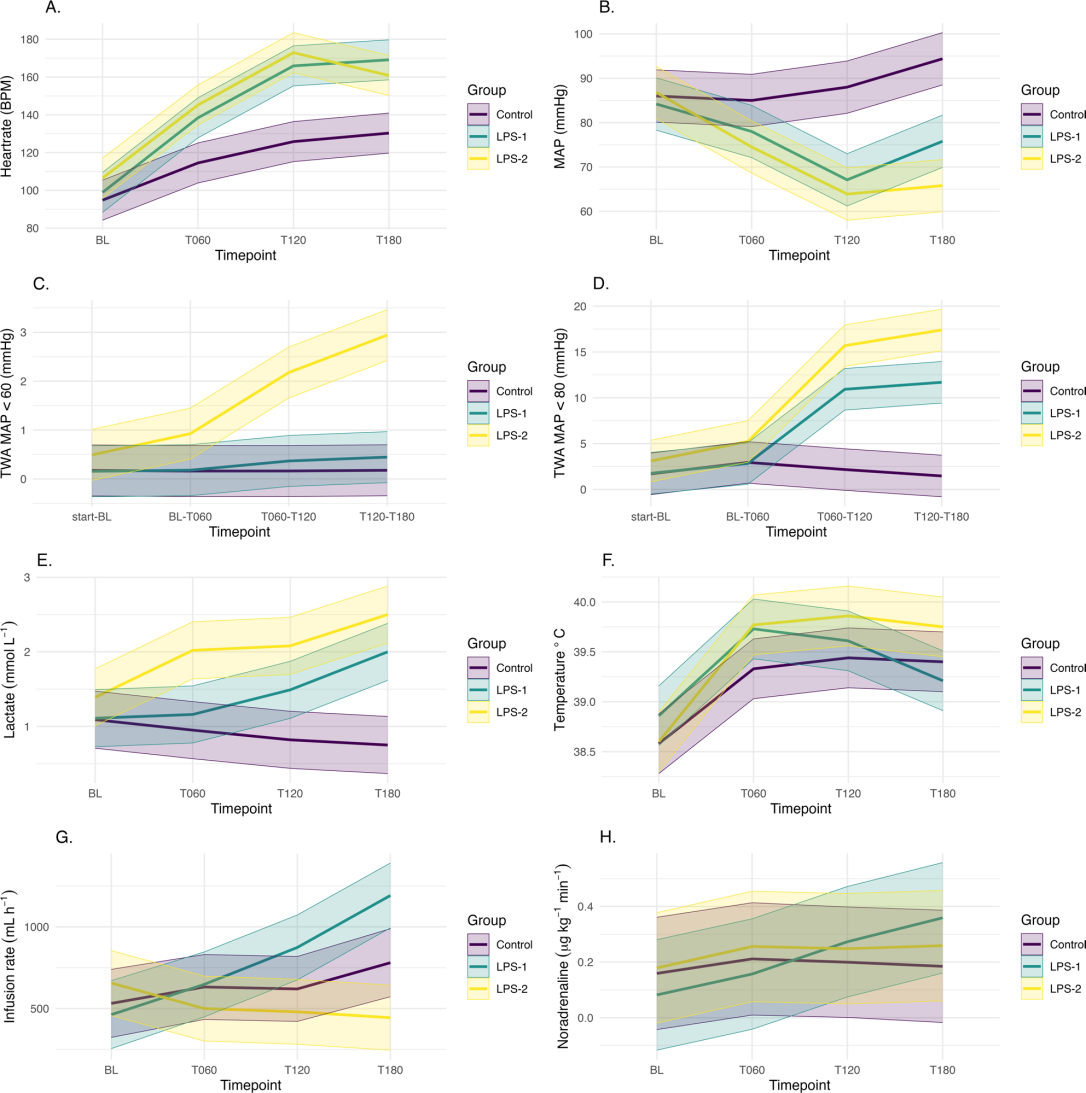
Overview monitored parameters effect plots; (**A**) Heartrate, (**B**) MAP, (**C**) TWA-MAP <60, (**D**) TWA-MAP <80, (**E**) Lactate, (**F**) Temperature, (**G**) Crystalloid infusion rate, and (**H**) Noradrenaline rate. LPS, lipopolysaccharide; LPS-1, LPS with support initiated at MAP <80mmHg; LPS-2, LPS with support initiated at MAP <65mmHg; MAP, mean arterial pressure; TWA-MAP, Time weighted average mean arterial pressure

Median [IQR] values for monitored parameters are presented in Table 1. Heart rate increased over time in all groups, with a significant group-by-time interaction reflecting a greater increase in both LPS groups compared with controls. MAP also showed a significant group-by-time interaction, with lower values in both LPS groups. TWA-MAP < 80 mmHg was higher at 120 and 180 min in both LPS groups, while TWA-MAP < 60 mmHg was higher at 120 min in LPS-2 only (Fig. 2A–D; Additional file 5, Table 1).

**Table 1:** Overview monitored parameters

	Time point	Control (*N*=10)	LPS-1 (*N*=10)	LPS-2 (*N*=10)
Heartrate (BPM)	Baseline	93 [84 - 104]	99 [93 - 107]	110 [86 - 118]
	T060	118 [97 - 125]	140 [128 - 153]	136 [134 - 149]
	T120	129 [126 - 133]	166 [155 - 175]	168 [161 - 181]
	T180	133 [119 - 139]	169 [156 - 178]	162 [145 - 168]
MAP (mmHg)	Baseline	88 [82 - 95]	85 [79 - 91]	85 [82 - 93]
	T060	86 [79 - 90]	74 [72 - 87]	70 [67 - 84]
	T120	89 [84 - 94]	66 [60 - 70]	62 [60 - 68]
	T180	97 [93 - 102]	73 [72 - 75]	62 [59 - 74]
TWA-MAP <60 (mmHg)	Baseline	0.13 [0.07 - 0.28]	0.16 [0.07 - 0.18]	0.47 [0.17 - 0.75]
	T060	0.17 [0.08 - 0.21]	0.06 [0.04 - 0.27]	0.86 [0.27 - 1.38]
	T120	0.04 [0.00 - 0.07]	0.31 [0.16 - 0.39]	2.66 [1.48 - 2.85]
	T180	0.09 [0.01 - 0.31]	0.25 [0.12 - 0.56]	2.45 [1.30 - 3.51]
TWA-MAP <80 (mmHg)	Baseline	1.95 [1.06 - 2.09]	1.33 [0.73 - 2.52]	2.87 [2.12 - 4.43]
	T060	0.87 [0.52 - 3.85]	1.33 [0.66 - 3.23]	5.89 [2.22 - 7.16]
	T120	0.51 [0.31 - 1.02]	11.01 [8.48 - 14.43]	17.65 [12.64 - 19.72]
	T180	0.82 [0.43 - 1.92]	12.53 [8.24 - 14.13]	17.11 [13.38 - 21.20]
Lactate (mmol·L^-1^)	Baseline	1.1 [0.9 - 1.3]	1.0 [0.8 - 1.4]	1.2 [1.0 - 1.6]
	T060	0.9 [0.7 - 1.1]	1.2 [1.0 - 1.2]	1.7 [1.2 - 2.7]
	T120	0.8 [0.7 - 0.9]	1.4 [1.3 - 1.5]	1.7 [1.5 - 2.2]
	T180	0.7 [0.6 - 0.8]	1.8 [1.6 – 2.0]	2.0 [1.8 - 2.7]
Temperature (°C)	Baseline	38.5 [38.1 – 39.0]	38.9 [38.6 - 39.2]	38.5 [38.3 - 38.8]
	T060	39.5 [39.0 - 39.6]	39.6 [39.4 - 40.3]	39.9 [39.7 - 39.9]
	T120	39.4 [39.4 - 39.6]	39.6 [39.3 - 39.9]	39.8 [39.5 - 40.1]
	T180	39.5 [39.2 - 39.6]	39.4 [38.7 - 39.5]	39.8 [39.5 - 39.9]
Infusion rate (ml·h^-1^)	Baseline	400 [240 - 800]	480 [240 - 560]	600 [400 - 800]
	T060	440 [400 - 600]	620 [480 - 800]	420 [400 - 600]
	T120	560 [420 - 780]	880 [560 - 1080]	420 [280 - 600]
	T180	720 [640 - 960]	1100 [915 - 1460]	400 [240 - 560]
Noradrenaline (µg·kg^-1^·min^-1^)	Baseline	0.03 [0.01 - 0.08]	0.07 [0.05 - 0.11]	0.05 [0.04 - 0.17]
	T060	0.08 [0.05 - 0.12]	0.06 [0.05 - 0.17]	0.12 [0.05 - 0.24]
	T120	0.10 [0.06 - 0.19]	0.19 [0.08 - 0.28]	0.09 [0.05 - 0.19]
	T180	0.08 [0.08 - 0.15]	0.19 [0.15 - 0.41]	0.05 [0.01 - 0.14]

LPS, lipopolysaccharide; LPS-1, LPS with support initiated at MAP <80mmHg; LPS-2, LPS with support initiated at MAP <65mmHg; MAP, mean arterial pressure; TWA-MAP, Time weighted average mean arterial pressure; Median [Inter Quartile Range]

**Table 2:** MitoPO_2_ and mitoVO_2_

	Time point	Control (*N*=10)	LPS-1 (*N*=10)	LPS-2 (*N*=10)
**MitoPO**_**2**_ **(mmHg)**				
Epidermal	Baseline	70.7 [51.9 - 89.6]	64.7 [48.6 - 87.5]	84.4 [63.4 - 89.3]
	T060	53.5 [41.6 - 77.9]	50.2 [35.6 - 74.0]	77.3 [46.2 - 80.9]
	T120	62.3 [56.1 - 76.5]	33.3 [31.3 - 56.1]	68.8 [48.5 - 72.3]
	T180	66.1 [62.7 - 84.7]	49.1 [43.6 - 63.5]	65.8 [40.3 - 82.4]
Intestinal Serosa	Baseline	45.0 [38.1 - 53.3]	50.8 [42.5 - 58.4]	51.1 [45.2 - 59.6]
	T060	62.3 [36.6 - 67.9]	54.3 [42.9 - 70.8]	55.9 [37.2 - 70.0]
	T120	67.0 [53.6 - 75.0]	58.2 [39.4 - 66.6]	41.2 [30.8 - 56.2]
	T180	45.6 [37.7 - 56.7]	42.8 [30.0 - 67.5]	43.7 [35.6 - 55.8]
Liver	Baseline	64.9 [62.2 - 81.7]	72.1 [61.6 - 78.2]	71.8 [68.7 - 74.3]
	T060	73.5 [65.1 - 88.7]	76.8 [59.7 - 87.6]	64.9 [55.2 - 80.9]
	T120	76.5 [60.8 - 90.2]	71.5 [58.9 - 82.7]	50.8 [44.1 - 78.2]
	T180	74.5 [63.4 - 87.3]	77.4 [67.0 - 90.7]	76.4 [63.7 - 83.5]
Renal cortex	Baseline	72.0 [68.4 - 79.6]	69.1 [58.8 - 80.9]	75.0 [71.4 - 78.1]
	T060	73.0 [69.0 - 75.6]	78.5 [77.0 - 86.6]	96.7 [90.5 - 103.0]
	T120	72.3 [55.4 - 76.4]	91.2 [82.3 - 97.8]	90.1 [79.3 - 95.7]
	T180	74.3 [68.9 - 81.4]	84.2 [69.6 - 89.0]	78.6 [67.3 - 91.7]
**MitoVO**_**2**_**(mmHg·s**^**-1**^)				
Epidermal	Baseline	15.2 [11.8 - 18.9]	17.9 [13.6 - 23.1]	16.7 [15.1 - 17.5]
	T060	11.5 [9.3 - 18.7]	18.1 [9.8 - 19.5]	15.8 [5.4 - 19.5]
	T120	15.3 [9.6 - 21.9]	10.2 [6.2 - 15.1]	15.5 [11.2 - 18.0]
	T180	16.8 [11.7 - 22.1]	11.4 [8.6 - 14.5]	19.9 [15.8 - 23.5]
Intestinal Serosa	Baseline	21.1 [18.3 - 22.5]	20.2 [16.3 - 23.2]	18.5 [17.4 - 22.4]
	T060	17.9 [16.5 - 22.6]	21.2 [20.2 - 23.5]	19.9 [11.7 - 23.1]
	T120	22.0 [17.5 - 22.9]	22.5 [18.8 - 24.2]	17.4 [12.2 - 23.0]
	T180	21.9 [19.6 - 25.7]	17.2 [15.7 - 22.3]	20.7 [15.1 - 22.5]
Liver	Baseline	16.5 [13.7 - 19.4]	17.6 [14.6 - 21.8]	17.4 [16.2 - 22.0]
	T060	21.5 [18.7 - 23.2]	18.2 [16.5 - 19.0]	16.6 [15.5 - 23.1]
	T120	18.2 [15.5 - 21.6]	19.9 [15.5 - 22.2]	15.8 [9.3 - 17.9]
	T180	22.6 [19.0 - 26.0]	18.8 [13.3 - 19.9]	17.2 [12.3 - 22.7]
Renal cortex	Baseline	30.1 [28.9 - 37.4]	36.2 [29.6 - 37.9]	34.5 [30.4 - 37.8]
	T060	33.0 [28.0 - 34.4]	37.5 [33.1 - 41.9]	42.0 [36.2 - 43.5]
	T120	30.0 [27.8 - 33.4]	35.6 [34.2 - 38.0]	34.0 [27.9 - 39.0]
	T180	28.4 [26.0 - 31.5]	29.6 [27.9 - 33.3]	29.0 [26.1 - 35.0]

LPS, lipopolysaccharide; LPS-1, LPS with support initiated at MAP <80mmHg; LPS-2, LPS with support initiated at MAP <65mmHg; mitoPO_2_, mitochondrial oxygenation; mitoVO_2_, mitochondrial oxygen consumption; Median [Inter Quartile Range]

Arterial lactate showed a significant group-by-time interaction, with higher levels in LPS-2 at all time points and in LPS-1 at 120 and 180 min compared with controls. Body temperature increased over time in all groups, with a lower temperature observed in LPS-1 at 180 min compared with controls, while no difference was observed in LPS-2 (Fig. 2E–F; Additional file 5, Table 2).

Infusion rates differed between groups over time, with higher rates in LPS-1 and lower rates in LPS-2 at 180 min compared to controls. Noradrenaline requirements were increased in LPS-1, with no significant change observed in LPS-2 ( Fig. 2G–H; Additional file 5, Table 3).

**Table 3:** Sublingual microcirculation

	Time point	Control (*N*=10)	LPS-1 (*N*=10)	LPS-2 (*N*=10)
Total vessel density (mm/mm^2^)	Baseline	25.5 [23.4 - 26.8]	25.0 [23.9 - 26.0]	27.5 [27.0 - 28.0]
	T060	26.2 [25.8 - 26.6]	26.4 [24.0 - 26.5]	27.7 [26.2 - 28.6]
	T120	26.0 [24.5 - 27.1]	24.3 [23.5 - 26.0]	25.3 [24.0 - 27.3]
	T180	25.9 [25.5 - 27.9]	23.7 [21.9 - 25.6]	26.6 [23.8 - 27.6]
Functional capillary density (mm/mm^2^)	Baseline	23.1 [21.8 - 24.0]	23.1 [21.6 - 24.2]	25.1 [23.6 - 26.2]
	T060	23.9 [23.4 - 25.2]	23.3 [22.0 - 24.3]	25.0 [23.5 - 26.3]
	T120	24.6 [23.5 - 24.8]	22.1 [21.0 - 23.7]	21.8 [21.2 - 24.1]
	T180	23.7 [23.6 - 25.8]	22.0 [18.6 - 23.5]	23.8 [21.2 - 24.4]
Proportion of perfused vessel (% vessel)	Baseline	0.90 [0.89 - 0.93]	0.91 [0.90 - 0.93]	0.92 [0.92 - 0.93]
	T060	0.94 [0.91 - 0.95]	0.90 [0.89 - 0.91]	0.91 [0.89 - 0.93]
	T120	0.94 [0.92 - 0.95]	0.91 [0.90 - 0.92]	0.88 [0.87 - 0.89]
	T180	0.93 [0.91 - 0.94]	0.90 [0.87 - 0.94]	0.89 [0.88 - 0.90]
Red blood cell velocity (µm·s^-1^)	Baseline	285.8 [253.1 - 296.6]	287.3 [283.1 - 299.8]	294.2 [291.7 - 304.2]
	T060	313.0 [289.7 - 330.0]	267.3 [262.3 - 274.6]	266.3 [245.9 - 290.2]
	T120	312.0 [298.0 - 315.2]	273.0 [262.1 - 292.5]	249.8 [239.4 - 266.4]
	T180	297.9 [283.8 - 307.3]	263.4 [249.6 - 274.6]	261.3 [248.7 - 268.3]
Tissue red blood cell perfusion (µm·min^-1^)	Baseline	62.3 [59.7 - 65.0]	63.1 [59.1 - 70.3]	71.9 [66.8 - 79.9]
	T060	70.7 [68.1 - 72.1]	61.9 [54.2 - 67.2]	69.2 [56.9 - 77.1]
	T120	71.2 [68.0 - 72.6]	58.9 [54.7 - 62.6]	58.6 [54.5 - 61.3]
	T180	70.4 [56.7 - 78.8]	56.5 [47.0 - 58.8]	62.1 [57.0 - 68.3]

LPS, lipopolysaccharide; LPS-1, LPS with support initiated at MAP <80mmHg; LPS-2, LPS with support initiated at MAP <65mmHg; Median [Inter Quartile Range]

### In vivo measurements

#### MitoVO_2_

**Fig. 3:**
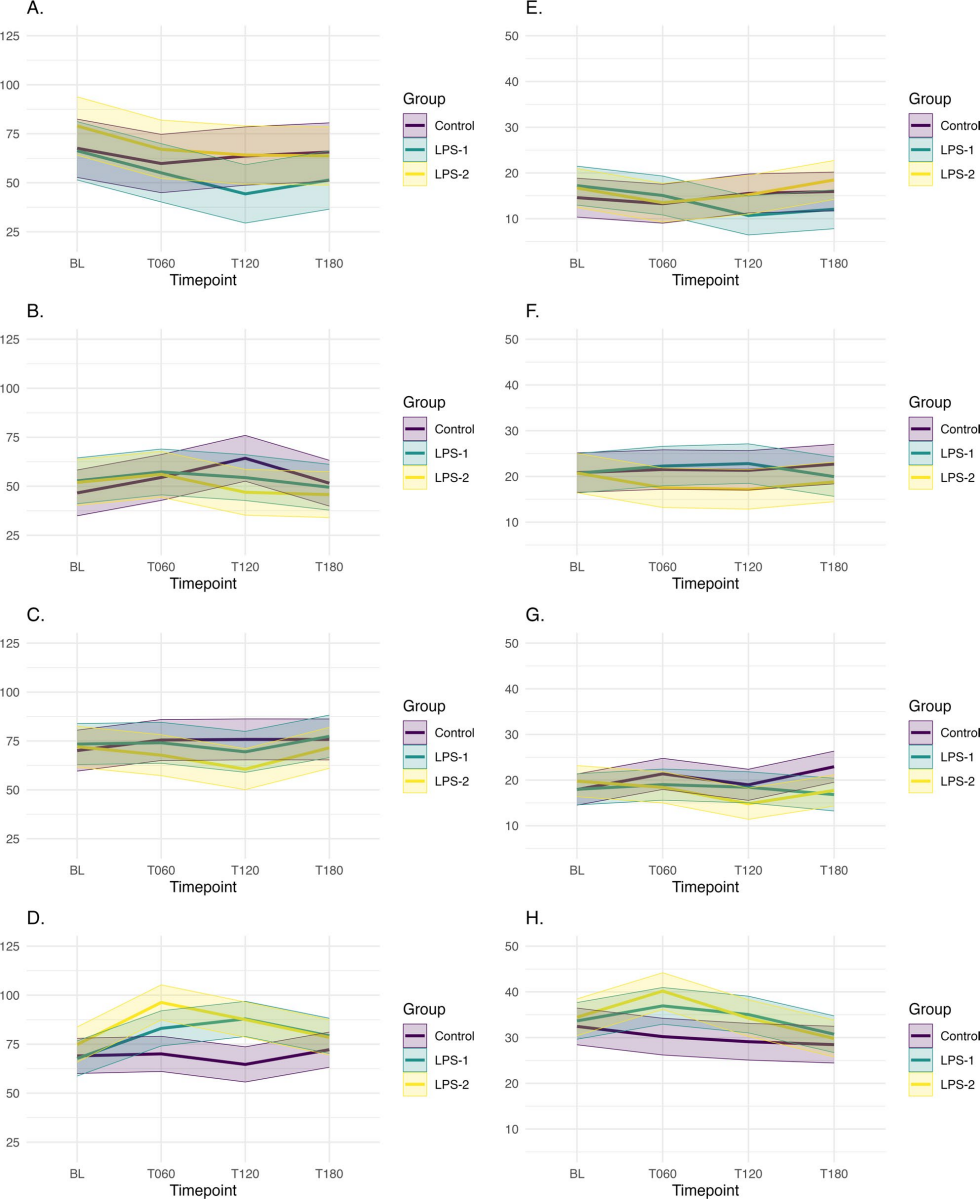
MitoPO_2_ and mitoVO_2_ effect plots; (**A**) Epidermal mitoPO_2_, (**B**) Intestinal serosa mitoPO_2_, (**C**) Liver mitoPO_2_, (**D**) Renal cortex mitoPO_2_, (**E**) Epidermal mitoVO_2_, (**F**) Intestinal serosa mitoVO_2_, (**G**) Liver mitoVO_2_, (**H**) Renal cortex mitoVO_2_. mitoPO_2_, mitochondrial oxygenation; mitoVO_2_, mitochondrial oxygen consumption; LPS, lipopolysaccharide; LPS-1, LPS with support initiated at MAP <80mmHg; LPS-2, LPS with support initiated at MAP <65mmHg

Detailed values are presented in Table 2. Epidermal mitoVO_2_ showed a significant group-by-time interaction, with lower values in LPS-1 at 120 and 180 min compared with controls, while no significant change was observed in LPS-2. No significant group-by-time interaction was observed for intestinal serosa mitoVO_2_ (Fig. 3E-F; Additional file 5, Table 4).

Liver mitoVO_2_ increased over time in controls, whereas this response was attenuated in both LPS groups, resulting in significantly lower values in LPS-1 at 180 min and in LPS-2 at 120 and 180 min compared with controls. Renal cortex mitoVO_2_ showed a transient group-by-time interaction, with higher values in LPS-2 at 60 min compared with controls, while no differences were observed at later time points or in LPS-1 (Fig. 3G-H; Additional file 5, Table 4).

#### MitoPO_2_

Median [IQR] values for mitoPO_2_ are shown in Table 2. Epidermal mitoPO_2_ remained stable over time with no significant group-by-time interaction. Intestinal serosal mitoPO_2_ showed a significant group-by-time interaction, with lower values in both LPS groups at 120 min compared with controls (Fig. 3B; Additional file 5, Table 5).

Liver mitoPO_2_ was largely preserved, although values were lower in LPS-2 at 120 min compared with controls. Renal cortex mitoPO_2_ showed a marked group-by-time interaction, with higher values in both LPS groups at 60 and 120 min compared with controls, while no differences were observed at 180 min (Fig. 3C-D; Additional file 5, Table 5).

#### Sublingual microcirculation

Median [IQR] values are presented in Table 3. TVD did not demonstrate a significant group-by-time interaction overall, although values were lower in LPS-2 at 120 min compared with controls. FCD showed a significant group-by-time interaction, with lower values in LPS-2 at 120 min and in both LPS groups at 180 min compared with controls.

**Fig. 4:**
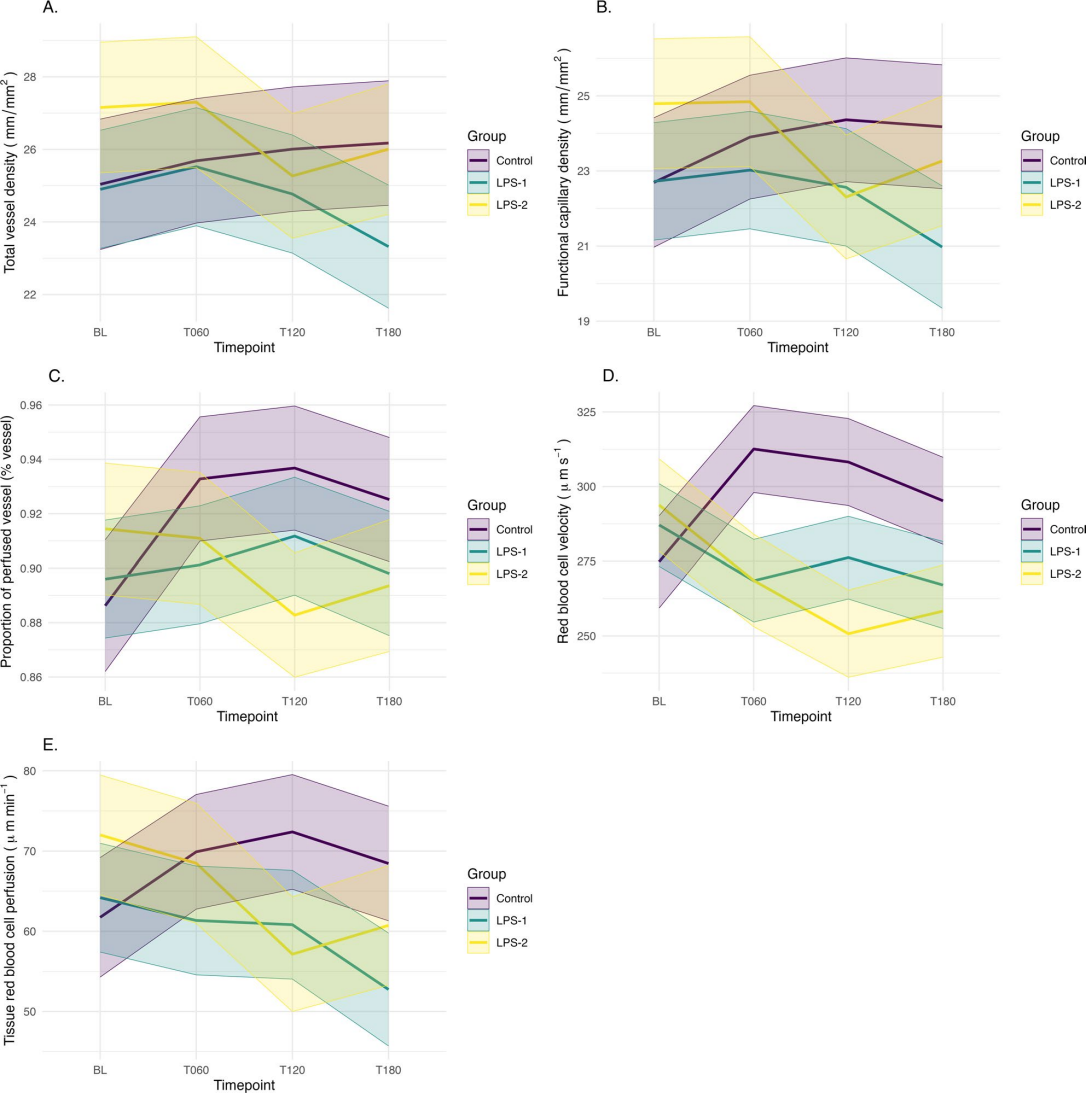
Sublingual microcirculation effect plots; (**A**) Total vessel density, (**B**) Functional capillary density, (**C**) proportion of perfused vessel, (**D**) Red blood cell velocity, (**E**) Tissue red blood cell perfusion. LPS, lipopolysaccharide; LPS-1, LPS with support initiated at MAP <80mmHg; LPS-2, LPS with support initiated at MAP <65mmHg

PPV also demonstrated a significant group-by-time interaction, increasing over time in controls but remaining lower in LPS-2 across all time points. RBC velocity and tissue RBC perfusion showed similar patterns, with increases at 60 and 120 min in controls and significant group-by-time interactions in both LPS groups, resulting in lower values compared with controls at all time points (Fig. 4A-E; Additional file 5, Table 6, and 7).

### Ex vivo measurements

Ex vivo measurements showed group-dependent differences. High-resolution respirometry demonstrated increased routine, leak, and maximal respiration in LPS-1, whereas changes in LPS-2 were limited. mtDNA and nDNA markers demonstrated modest alterations over time, including increased PBMC mtND1/GCG ratios in LPS-1and lower plasma mtND1/GCG ratios in both LPS groups. Plasma mtND1 concentrations did not change significantly, while plasma GCG concentrations were higher in both LPS groups at early time points. HIF-1α expression was minimal across all organs and did not differ between groups (Additional file 2).

### Organ function

Organ function markers showed group-dependent changes. Right kidney clearance was lower in LPS-2 at 120 min compared with controls. NGAL concentrations were higher at 180 min, particularly in LPS-2 and to a lesser extent in LPS-1. ICG-PDR decreased over time in controls and was further reduced in both LPS groups, with an inverse correlation between ICG-PDR and arterial lactate concentrations. I-FABP increased over time in controls, with no differences observed between LPS groups (Additional file 3).

## Discussion

### LPS model

This model employed two LPS groups defined by different MAP thresholds for initiation of hemodynamic support, alongside a control group, to study early endotoxemia under standardized hemodynamic support with noradrenaline and crystalloid fluids. LPS administration was associated with pronounced hemodynamic stress, evidenced by increased heart rate and reduced MAP compared with controls, alongside elevated arterial lactate levels, which were earlier and more sustained in LPS-2, indicating a systemic stress response during early endotoxemia.

Differences between the LPS groups were not always apparent, as MAP largely reflected the threshold-based initiation of hemodynamic support rather than fixed pressure targets. Accordingly, differences between groups were more readily captured by cumulative measures such as TWA-MAP, which demonstrated a greater hypotension burden in LPS-2 and modest but consistent separation in cumulative hypotension exposure between the LPS groups during early endotoxemia. This approach was intended to generate graded hypotension exposure rather than maintain fixed MAP levels.

A gradual increase in heart rate was also observed in the control group over time, likely reflecting ongoing surgical stress related to organ exposure and instrumentation.

### In vivo measurements

The primary objective of this study was to characterize the dynamics of mitoVO_2_ during early endotoxemia and resuscitation. In the epidermis, mitoVO_2_ was lower in LPS-1 at 120 and 180 min compared with controls, while epidermal mitoPO_2_ remained unchanged across all groups and time points. Notably, this reduction was not observed in LPS-2, which may appear counterintuitive given the greater hypotension burden in this group. In the liver, mitoVO_2_ increased over time in controls but this response was blunted in both LPS groups, resulting in lower values at later time points (at 180 min in LPS-1, and at 120 and 180 min in LPS-2). Liver mitoPO_2_ remained largely stable in LPS-1, with a transient reduction at 120 min in LPS-2.

This apparent discrepancy between hypotension burden and mitoVO_2_ response should be interpreted with caution, as separation between the LPS groups was modest and primarily reflected cumulative exposure rather than consistent differences in instantaneous MAP. In the early phase of endotoxemia, physiological responses are highly dynamic and regionally heterogeneous, which may have limited detection of a clear relationship between hypotension and mitoVO_2_ [31].

Taken together, these findings suggest a partial uncoupling between mitochondrial oxygen availability (mitoPO_2_) and mitochondrial oxygen utilization (mitoVO_2_) during early endotoxemia, most evident in the epidermis, and to a lesser extent, in the liver due to the blunted time course. This interpretation aligns with previous work from Harms et al., showing impaired mitochondrial oxygen utilization despite preserved mitoPO_2_ [15]. Accordingly, maintaining mitoPO_2_ alone may not reliably reflect mitochondrial metabolic function, with this relationship appearing tissue- and context-specific.

Given the alterations in mitoVO_2_, sublingual microcirculation was assessed to further characterize early endotoxemia. LPS-induced endotoxemia resulted in reduced FCD, RBC velocity, and tissue RBC perfusion in both LPS groups, with an additional reduction in PPV in LPS-2. While PPV increased over time in controls, it remained lower in LPS-2, indicating greater microcirculatory disturbance under more permissive hypotension.

De Backer et al. identified PPV as a strong predictor of mortality in severe sepsis, with lower PPV observed in patients assessed early after ICU admission compared with later time points [32]. In contrast, the microcirculatory disturbances observed in the present study were relatively modest and occurred within a short, 3-hour observation period. This limited timeframe, combined with early hemodynamic resuscitation, may explain why more pronounced or progressive microvascular derangements, such as those described in clinical sepsis, were not observed. Recently, Flick et al. demonstrated impaired intestinal microvascular flow and increased flow heterogeneity during porcine LPS-induced endotoxic shock, with recovery after sequential fluid and norepinephrine resuscitation [33]. Although direct comparison is limited by differences in measurement site, hemodynamic targets, and resuscitation strategy, these findings further support that microcirculatory responses during endotoxemia are measuring site- and context-dependent. Specifically, their interpretation depends on the scope of measurement, whether targeting tissue perfusion or mitochondrial oxygenation, and on the nature and severity of the underlying pathophysiological insult.

Importantly, microcirculatory impairment in this model was modest, with a maximal ~ 19% reduction in tissue RBC perfusion in LPS-2. Although these measurements were obtained from the oral mucosa, they are consistent with the mathematical framework described by Hilderink et al., which demonstrated that only substantial reductions in microvascular flow velocity or vessel density (on the order of ~ 50%) are expected to meaningfully affect mitoPO_2_. Smaller reductions fall within a plateau region of the microcirculation-mitoPO_2_ relationship, owing to its logarithmic nature [34]. This likely explains the largely preserved mitoPO_2_ despite measurable microcirculatory changes.

### Ex vivo mitochondrial function and organ responses

During early endotoxemia, PBMC mitochondrial respiration showed a transient increase in routine and maximal respiration, suggesting a hyperdynamic and potentially adaptive early-phase response rather than overt mitochondrial dysfunction (Additional file 2). In contrast, mitoVO_2_ appeared blunted in selected organs, highlighting a discrepancy between preserved ex vivo respiratory capacity and constrained in vivo oxygen utilization. Organ function analyses indicated early signs of hepatic and renal impairment, reflected by reduced ICG-PDR, elevated lactate likely partly due to impaired clearance, and increased urinary NGAL, particularly under greater hypotension exposure (Additional file 3). These findings underscore the dynamic and context-dependent nature of mitochondrial and organ responses during early endotoxemia, where systemic and microenvironmental factors critically modulate functional outcomes.

### Integrative perspective

MitoPO_2_ remained largely preserved despite reductions in red blood cell perfusion in the LPS groups, suggesting that early microcirculatory disturbances did not substantially limit mitochondrial oxygen availability. This is consistent with prior work indicating that more pronounced microvascular impairment is required before mitoPO_2_ declines appreciably [34].

In vivo tissue measurements showed reduced or blunted mitoVO_2_ in selected organs, whereas ex vivo PBMC respirometry demonstrated increased respiratory activity. Together with elevated lactate levels, this indicates a complex and potentially discordant relationship between oxygen availability, mitochondrial utilization, and systemic metabolic markers during early endotoxemia. Importantly, preserved mitoPO_2_ does not necessarily reflect preserved mitochondrial metabolic function at the tissue level.

These results underscore the limitations of relying on single physiological or biochemical parameters, such as lactate concentration, microcirculatory indices, or global hemodynamic targets, to infer tissue oxygen utilization or cellular metabolic state. An integrated assessment incorporating mitoPO_2_, mitoVO_2_, microcirculatory perfusion, and systemic hemodynamics may provide a more complete representation of tissue-level oxygen handling during endotoxemia. Such an approach may help contextualize resuscitation responses and avoid overinterpretation of isolated signals, particularly during the early, hyperdynamic phase of sepsis, where conventional markers may not reliably reflect underlying cellular metabolic requirements.

### Strengths and limitations

Animal models are essential to understand the systemic effects of sepsis and septic shock, as disease progression cannot be standardized in humans [35], with swine representing a highly translational model due to their physiological similarities to humans and pronounced response to LPS [19, 20]. The use of SPF swine improved reproducibility by minimizing variability associated with pathogen exposure, although this may also have reduced LPS tolerance [36]. Together with a standardized experimental setup and the combined assessment of in vivo and ex vivo mitochondrial function alongside microcirculatory and organ measurements, this approach provides an integrated insight into early LPS-induced endotoxemia.

Despite these strengths, several limitations warrant consideration. Although the study design aimed to create differences in MAP using two different MAP thresholds for initiation of hemodynamic support, this approach was intended to generate graded hypotension exposure rather than maintain fixed MAP levels. Clear separation in instantaneous MAP was not consistently achieved. While TWA-MAP metrics indicated differences in cumulative hypotension, substantial overlap in instantaneous MAP values likely reflects the dynamic nature of early endotoxemia and the need for pragmatic hemodynamic management. In addition, total fluid volumes were similar across groups despite differences in infusion rates. This was likely due in part to relative hypovolemia after the 12-hour fasting period, necessitating substantial fluid administration before baseline [37]. Fasting may also have influenced baseline metabolic state and early metabolic responses [38]. However, as fasting conditions were standardized across all animals, this is unlikely to have affected between group comparisons, although it may have contributed to variability in MAP and early fluid requirements.

Another limitation is the short study duration, with only 3 h of observation after LPS administration, restricting analysis to the early hyperdynamic phase. This may have precluded detection of later hemodynamic, inflammatory, and mitochondrial changes, including the development of endotoxin tolerance, which has been reported to emerge within 6–12 h [39–41].

Finally, translating findings from an LPS model to clinical sepsis remains challenging. Human sepsis develops over a longer time course, often involves live pathogens and comorbidities [8], and differs from controlled endotoxin exposure. Moreover, LPS dosing varies across models [39, 42, 43]; in this study, doses were chosen to establish an ICU-relevant endotoxemia model, rather than induce a maximal inflammatory response.

## Conclusion

This study shows that early LPS-induced endotoxemia, managed with two distinct MAP thresholds for initiation of hemodynamic support, is associated with measurable physiological and metabolic alterations. mitoVO_2_ was altered in selected tissues, while mitoPO_2_ was largely preserved or only transiently affected in those tissues, suggesting that mitochondrial oxygen availability and utilization do not necessarily change in parallel during early endotoxemia. Microcirculatory disturbances were present but modest, such that changes in mitochondrial function occurred in a context of relatively preserved microvascular perfusion.

Together, these findings highlight the complexity of early endotoxemia, in which apparently adequate systemic hemodynamics and preserved mitochondrial oxygen availability do not necessarily ensure preserved tissue-level mitochondrial function. Future studies are warranted to evaluate later-stage responses and their implications for resuscitation strategies.

Wijnie van Dam, MSc, Laboratory of Translational Intensive Care, Department of Intensive Care Adult, Erasmus MC, Rotterdam, the Netherlands, facilitated the sublingual microcirculation measurements.

## Supplementary Information

Below is the link to the electronic supplementary material.

Supplementary Material 1

Supplementary Material 2

Supplementary Material 3

Supplementary Material 4

Supplementary Material 5

## Data Availability

The datasets used and/or analyzed during the current study are available from the corresponding author on reasonable request.

## Abbreviations

ABP: Arterial blood pressure
ALA: 5-aminolevulinic acid
CCO: Continuous cardiac output
CVP: Central venous pressure
ECG: Electrocardiogram
FCD: Functional capillary density
GCG: Glucagon
HR: Heart rate
ICG: Indocyanine green
ICG-PDR: Indocyanine green plasma disappearance rate
ICU: Intensive care unit
IDF: Incident dark-field
I-FABP: Plasma intestinal fatty acid binding protein
IQR: Interquartile range
LMM: Linear mixed model
LPS: Lipopolysaccharide
MAP: Mean arterial pressure
mitoPO_2_: Mitochondrial oxygen tension
mitoVO_2_: Mitochondrial oxygen consumption
mtDNA: Mitochondrial DNA
mtND1: NADH Dehydrogenase 1
nDNA: Nuclear DNA
NGAL: Neutrophil gelatinase-associated lipocalin
OCR: Oxygen consumption rate
ODR: Oxygen disappearance rate
PaO_2_: Arterial oxygen tension
PBMCs: Peripheral blood mononuclear cells
PpIX: Protoporphyrin-IX
PpIX-TSLT: Protoporphyrin-IX-triplet lifetime state technique
PPV: Proportion perfused vessels
RBCv: Red blood cell velocity
RDM: Rectangular distribution method
SD: Standard deviation
SPF: Specific pathogen free
SpO_2_: Oxygen saturation
tRBCp: Tissue red blood cell perfusion
TVD: Total vessel density
TWA-MAP: Time weighted average-mean arterial pressure
