# Supplementary material for: In vivo mitochondrial oxygen consumption during LPS-induced endotoxemia: a controlled experimental study in swine
Source: Intensive Care Med Exp. 2026 Jul 20;14:97. doi: 10.1186/s40635-026-00951-z (PMC13385532; doi:10.1186/s40635-026-00951-z)
Supplement: Supplementary file 1 — Supplementary Material 1 [file 40635_2026_951_MOESM1_ESM.docx]

**Additional file 1**: *In vivo* mitochondrial measurements

*Overview*

This supplement describes the methodology used for *in vivo* assessment of mitochondrial oxygen tension (mitoPO_2_) and mitochondrial oxygen consumption (mitoVO_2_) based on delayed fluorescence lifetime measurements of protoporphyrin IX (PpIX). The principles of the optical measurement technique, ALA administration, data acquisition, and analysis procedures are detailed below.

*ALA administration and preparation of the measurement site*

To enable mitoPO_2_ and mitoVO_2_ measurements on the skin, the anterior interscapular area was first shaved to remove hair. The skin was then prepared by gently rubbing it with fine sandpaper and cleaning it with ethanol (70%) gauze to remove the top layers of the stratum corneum. Freshly prepared 5-aminolevulinic acid (ALA) cream (20%, 2 g per 3 x 3 cm area; Carl Roth GmbH & Co. KG, Karlsruhe, Germany) was applied to the skin, covered with IV3000 plaster (Smith & Nephew, United Kingdom), and shielded from ambient light using aluminum foil.

Following establishment of femoral venous access at the start of the experiment, ALA was also administered intravenously (2.5 mg), dissolved in Dulbecco’s Phosphate Buffered Saline (DPBS; Biowest, Nuaillé, France) to a final concentration of 1 mg•mL^-1^ with pH adjusted to 7.4. After completion of all surgical preparations 3 hours after ALA administration, when sufficient protoporphyrin-IX (PpIX) synthesis had occurred, baseline measurements were initiated. After baseline measurements, a second dose of ALA (1.5 mg, same concentration and pH) was administered to maintain adequate signal quality during subsequent measurements.

*Optical and laser setup*

The optical setup and excitation detection principles used in this study were identical to those described in earlier methodological work and were not modified for the present experiments [1]. Briefly, PpIX excitation was achieved using a pulsed, tunable laser system coupled via optical fibers to a custom reflection probe. Delayed fluorescence emission was detected using a gated photomultiplier tube, with appropriate spectral filtering to isolate the PpIX signal.

Excitation pulses had a duration in the nanosecond range and an energy of approximately 250 μJ per pulse. Measurements were performed at a repetition frequency of 1 Hz, corresponding to one laser pulse and one mitoPO_2_ determination per second. A detailed description of the laser system, fiber optics, detection hardware, and optical filtering has been published previously and is referenced here for completeness [1].

***Principle of mitoPO_2_ measurement using PpIX delayed fluorescence***

**PpIX possesses a triplet excited state whose lifetime is strongly quenched by molecular oxygen, making the delayed fluorescence lifetime oxygen dependent. Upon photoexcitation with a short laser pulse, PpIX emits red delayed fluorescence. The decay time (lifetime, τ) of this emission is inversely related to the local oxygen tension [1, 2].**

**mitoPO_2_ is derived from the measured delayed fluorescence lifetime according to the Stern-Volmer relationship:**

$$PO_{2}= \frac{\frac{1}{\tau}-\frac{1}{\tau_{0}}}{k_{q}}$$

**where τ is the measured delayed fluorescence lifetime, τ_0_ is the lifetime at zero oxygen, and k_q_ is the oxygen quenching constant (in mmHg^-1^s^-1^) [2].**

**Because mitochondria within the measurement volume exhibit intrinsic spatial heterogeneity in oxygen tension, mitoPO_2_ is not a single uniform value. To robustly estimate a representative mean mitoPO_2_ under these non-homogeneous conditions, the Rectangular Distribution Method (RDM) is applied. The RDM assumes a rectangular distribution of oxygen tensions within the sampled volume and yields a stable estimate of the mean mitoPO_2_ that is less sensitive to noise and heterogeneity than mono-exponential fitting approaches [3].**

A batch processing tool was developed using LabView 2018 (National Instruments, Austin, TX, USA) to apply the RDM fit to mitoPO_2_ laser data recorded in LabView 2013. This tool enabled batch processing of individual laser measurement files, streamlining data analysis. Minor adjustments were made to the to fully integrate the batch processing functionality for RDM fitting.

***mitoVO_2_ measurement: oxygen disappearance rate (ODR)***

mitoVO_2_ was assessed *in vivo* using the ODR technique. This approach is based on transient cessation of microvascular oxygen supply combined with continued cellular oxygen consumption [1].

During mitoVO_2_ measurements, the reflection probe was pressed against the measurement site to locally occlude the microcirculation, thereby creating stop-flow conditions. Under these conditions, oxygen delivery is temporarily halted, and the ongoing mitochondrial oxygen consumption results in a measurable decline in mitoPO_2_ over time.

MitoPO_2_ was recorded at a sampling frequency of 1 Hz throughout the measurement sequence, using one laser pulse per data point. The resulting mitoPO_2_ time curve typically consists of an initial stable phase followed by a linear decline during stop-flow conditions.

*mitoVO_2_ analysis*

For mitoVO_2_ analysis, mitoPO_2_ values were retrieved programmatically from the recorded datasets. The portion of the mitoPO_2_ time curve corresponding to linear oxygen decline during stop-flow was identified, and a linear fit was applied to this segment. The slope of this fitted line represents the maximal ODR (Vmax), expressed in mmHg•s^-1^, which is reported as mitoVO_2_, an example is shown in **Fig**. 1.

Curve fitting was performed using a custom Dash-based linear Vmax analysis tool that enabled visual inspection and fitting of individual curves. To ensure blinded analysis, filenames were pseudonymized prior to fitting, obscuring group allocation and subject identity.

In a minority of measurements, mitoVO_2_ could not be reliably determined due to artifacts or absence of a clear linear decline. Specifically, fitting was not possible in approximately 3% of measurements; 21 measurements were excluded due to motion or signal artifacts, and 28 lacked a clearly discernible mitoVO_2_ curve.


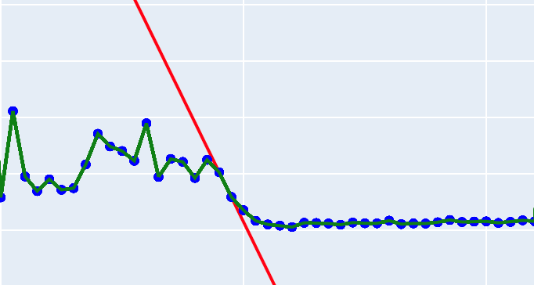

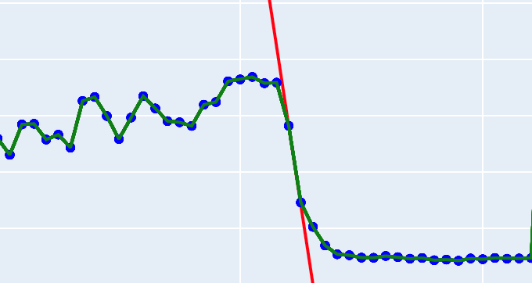


**Fig 1.** Example of linear fitting on mitoVO_2_ curves

**References**

1. Harms FA, Bodmer SI, Raat NJ, Mik EG. Non-invasive monitoring of mitochondrial oxygenation and respiration in critical illness using a novel technique. Crit Care. 2015;19(1):343.

2. Bodmer SI, Balestra GM, Harms FA, Johannes T, Raat NJ, Stolker RJ, et al. Microvascular and mitochondrial PO(2) simultaneously measured by oxygen-dependent delayed luminescence. J Biophotonics. 2012;5(2):140-51.

3. Harms FA, de Boon WM, Balestra GM, Bodmer SI, Johannes T, Stolker RJ, et al. Oxygen-dependent delayed fluorescence measured in skin after topical application of 5-aminolevulinic acid. J Biophotonics. 2011;4(10):731-9.
