# Supplementary material for: In vivo mitochondrial oxygen consumption during LPS-induced endotoxemia: a controlled experimental study in swine
Source: Intensive Care Med Exp. 2026 Jul 20;14:97. doi: 10.1186/s40635-026-00951-z (PMC13385532; doi:10.1186/s40635-026-00951-z)
Supplement: Supplementary file 2 — Supplementary Material 2 [file 40635_2026_951_MOESM2_ESM.docx]

**Additional file 2:** *Ex vivo* measurements

**1. Methods**

1.1 High-resolution respirometry

*PBMC isolation*

Arterial blood samples were collected (BD Vacutainer^®^ K2EDTA) for peripheral blood mononuclear cell (PBMC) isolation and mitochondrial function measurements. For BL and T060, 10 mL of blood was collected from all groups. At T120 and T180, additional blood was required due to PBMC loss or blood dilution: 20 mL (2 × 10 mL) in the LPS-1 group and 30 mL (3 × 10 mL) in the LPS-2 group. PBMC isolation from blood samples at BL, T060, T120, and T180 began immediately or within 1 hour after collection. All procedures were performed at room temperature.

PBMC isolation was performed following a modified version of the MiPNet 21.17 blood cell isolation protocol. Briefly, 5 mL of blood was added to a Leucosep™ tube 12 mL (Greiner Bio-One, Frickenhausen, Germany) containing Lymphoprep™ (Biovision, Milpitas, CA, USA) as the separation medium. The blood was diluted with DPBS to a final volume of 12 mL, gently mixed, and centrifuged at 800 × g for 20 minutes. The buffy coat was collected with a Pasteur pipette, and the isolated PBMCs were washed twice in 14 mL DPBS and centrifuged at 250 × g for 10 minutes.

After the final wash, the pellet was resuspended in 5 mL RPMI-1640 medium (Gibco, Paisley, UK). For later DNA isolation, 500 μL of the PBMC suspension was removed and washed twice in phosphate-buffered saline. The PBMC pellet was snap frozen and stored at -80°C. The remaining PBMC suspension was used for high-resolution respirometry analysis using the Oxygraph O2k (Oroboros Instruments, Innsbruck, Austria).

*Oroboros calibration*

Prior to cell suspension loading, a volume calibration was performed, and the instrument was calibrated following manufacturer instructions with 1.5 mL RPMI-1640 medium. An oxygen solubility factor of 0.89 was used to calculate oxygen levels in RPMI-1640 medium. After calibration, the RPMI-1640 medium was removed from the chamber and replaced with 2.1 mL PBMC suspension.

During analysis of the OCR, the chamber was closed and equilibrated, and a coupling-control protocol was applied to assess mitochondrial function. Definitions of the mitochondrial respiration states were based on Gnaiger et al. Respiration states were measured at 37°C with a stirring speed of 750 rpm, and experiments were conducted at O_2_ concentrations >50 µM to prevent oxygen-dependent respiration [1, 2].

*Oroboros chemicals*

All chemicals for the mitochondrial experiments were purchased from Sigma-Aldrich (St. Louis, MO, USA). Oligomycin was added to the chamber (0.01 mM stock; 2 µL), effectively blocking ATP synthase activity to measure non-ATP-linked (leak) respiration. Oligomycin concentration tests were performed to obtain this optimum concentration of Oligomycin, because high concentrations of Oligomycin can decrease the ET-capacity owing to its uncoupling side effect. Subsequently, serial additions of the uncoupler carbonyl cyanide p-trifluoromethoxy phenylhydrazone (FCCP) (0.25 mM stock in steps of 0.5 µL) were added until a maximal (max) respiration rate was obtained. FCCP additions were continued until 1–2 consecutive additions failed to increase the respiration rate. About 2–8 injections of 0.5 µL of a 0.25 mM FCCP were given, resulting in end concentrations of 0.125 µM to 0.5 µM FCCP. The specific complex I inhibitor rotenone (1 mM stock; 1 µL) and, finally, the complex III inhibitor antimycin A (5 mM stock; 1 µL) were added for non-mitochondrial respiration, which is independent of electron transfer chain activity. This residual oxygen consumption (ROX), which is not affected by these inhibitors is attributable to other cellular oxygen-consuming processes than the mitochondrial respiratory chain.

*Oxygen flux*

Oxygen flux was quantified using DatLab software (version 5, OROBOROS Instruments, Innsbruck, Austria) and ROX was subtracted from routine, leak, and max OCRs for the evaluation of oxygen consumption specifically attributable to mitochondrial respiration. When values after subtraction were below zero, the values were set to zero. The final PBMC concentration in the chamber was measured with an automated hematology analyzer (XN-10, Sysmex^®^, Kobe, Japan). The OCRs were corrected for cell concentration.

The routine, leak and max OCRs were determined for the PBMCs.

1.2 mtDNA and nDNA

EDTA plasma was thawed and filtered using a SpinX 0.22 µm filter (16,000g for 2 min) (#8160, Corning Costar, Salt Lake City, UT, USA). 100 µL of the filtrate was used for DNA isolation [3]. DNA isolation was performed using the Qiagen DNeasy Blood & Tissue kit (#69504, Qiagen, Hilden, Germany). The process was automated by the QiaCube Connect (Qiagen, Hilden, Germany), and DNA was eluted in 150 µL AE buffer.

The PBMC pellets derived from the steps detailed in Additional file 2 were thawed and 100 µL phosphate buffered saline was added to the sample. DNA isolation followed the same procedure as above, with DNA eluted in 200 µL AE buffer.

mtDNA and nDNA were analyzed using the following primers:

- mtDNA: Swine NADH Dehydrogenase 1 (mtND1), forward primer: 5’-CAGGCACATCCTCAATCTCC-3’, reverse primer: 5’-CCCCGATGAGTGCGTATTTT-3’.
- nDNA: Swine Glucagon (GCG), forward primer: 5’-GTCTTCACATCATAAAGCAGATGTC-3’, reverse primer: 5’-AGAGAAGTTCGTGGCAAACAC-3’.

A final primer concentration of 400 nM was used for all reactions [3].

To quantify mtDNA (mtND1) and nDNA (GCG) levels, a positive control with a known copy number was included as a 10-fold dilution series on every plate. The positive control consisted of a synthetic dsDNA fragment (gBlock, IDT Integrated DNA Technologies, Leuven, Belgium).

Quantitative PCR (qPCR) was performed using the SensiMix SYBR & Fluorescein kit (#QT615-05, Bioline, Meridian Bioscience, Memphis, Tennessee) in combination with Bio-Rad CFX96 and Bio-Rad CFX Opus 384 real-time systems (Bio-Rad Laboratories, California, USA). All samples and standards were measured in triplicate, and a "no template control" (negative control) was included. The qPCR program consisted of 2 min at 50 °C, followed by 10 min at 95 °C, and then 40 cycles of 15 sec at 95 °C and 1 min at 58 °C. A melting curve analysis was performed at the end to verify amplification specificity. Data were analyzed using Bio-Rad CFX Maestro 2.3 software (Bio-Rad Laboratories, California, USA).

After centrifuge 1000g for 10 min, EDTA plasma DNA samples were used undiluted in a 96-well format. To convert DNA copy numbers (copies•µL⁻¹) into plasma concentrations (copies•µL⁻¹ plasma), the following formula was used:

$$c = Q \times\left( \frac{Vdna}{Vpcr} \right)\times\left( \frac{1}{Vext} \right)$$

Where:

- **c** = copies/µL^-1^ plasma
- **Q** = copies calculated by qPCR software
- **Vₐdna** = volume of extracted DNA (150 µL from the final step of DNA isolation)
- **Vₐpcr** = volume of DNA used for qPCR (10 µL)
- **Vₑext** = volume of plasma used for DNA isolation (100 µL plasma)

PBMC DNA samples were diluted 1:5 and processed in a 384-well format. Mitochondrial content was calculated by dividing the quantity of mtDNA by the quantity of nDNA, resulting in the mtND1/GCG ratio.

1.3 Histopathology

At the end of the experiment, tissue sections from the kidney, liver and intestine were fixed in 3.8-4.2% buffered formaldehyde (Boom B.V., Meppel, The Netherlands) for at least 24 hours. The fixed tissues were then dehydrated in ethanol, cleared in xylene, and embedded in paraffin. Thin sections (5 µm) were prepared for HIF-1α immunostaining.

HIF-1α accumulation in pig kidney, intestine, and liver sections (n = 8-9 per group) was assessed by immunohistochemical staining using an HIF-1α rabbit polyclonal antibody (1:300 dilution; Novus Biologicals, Littleton, CO). After deparaffinization and rehydration, antigen retrieval was performed by heating the sections in sodium citrate buffer (pH 6.0) for 15 minutes. Sections were then blocked in 3% H_2_O_2_-methanol for 20 minutes at room temperature and permeabilized with Tris-buffered saline containing 0.5% Tween (TBS-T) for 20 minutes.

To prevent non-specific binding, sections were incubated with 5% normal goat serum in TBS-T (0.05%) followed by overnight incubation with the anti-HIF-1α antibody at 4°C. Detection was carried out using the Dako EnVision Detection System, Peroxidase/DAB+ Rabbit/Mouse, with hematoxylin counterstaining. After dehydration with ethanol and xylene, the sections were prepared for mounting.

Images were captured using the NanoZoomer (Hamamatsu Photonics, Shizuoka, Japan). Clear brown-yellow staining, restricted to the cytoplasm, nuclei, or cell membrane, indicated a positive HIF-1α result. Only nuclear staining was considered positive for hypoxia, and this was quantified using QuPath 5.1 software.

**2. Results**

2.1 High-resolution respirometry

The median [IQR] values are presented in Table 1. Routine respiration demonstrated a significant group-by-time interaction. Routine respiration remained stable in the control group but was higher in LPS-1 (LPS with hemodynamic support initiated at MAP <80 mmHg) at all time points and in LPS-2 (LPS with hemodynamic support initiated at MAP <65 mmHg) at 180 minutes compared with controls. Leak respiration remained stable in controls and LPS-2 but was higher in LPS-1 across all time points. Maximal respiration increased significantly over time in controls. A significant group-by-time interaction was observed in the LPS-1, with higher values at 120 and 180 minutes, while no significant group-by-time interaction was observed in LPS-2 (Fig. 1A–C; Table 2).

**Table 1:** High-resolution respirometry

|  | Time point | Control (N=10) | LPS-1 (N=10) | LPS-2 (N=10) |
| --- | --- | --- | --- | --- |
| *Routine (pmol/(s*10^6^ PBMCs))* | Baseline | 2.43 [2.33 - 2.50] | 2.70 [2.33 - 2.96] | 2.99 [2.44 - 3.22] |
|  | T060 | 2.76 [2.65 - 3.42] | 5.40 [3.40 - 8.32] | 4.59 [3.86 - 6.54] |
|  | T120 | 3.02 [2.54 - 4.01] | 5.76 [4.78 - 7.05] | 4.29 [3.69 - 5.45] |
|  | T180 | 3.61 [3.30 - 3.79] | 6.99 [4.62 - 8.99] | 5.56 [4.23 - 6.79] |
| *Leak (pmol/(s*10^6^ PBMCs))* | Baseline | 0.43 [0.37 - 0.47] | 0.54 [0.43 - 0.61] | 0.57 [0.43 - 0.72] |
|  | T060 | 0.55 [0.46 - 0.86] | 1.64 [1.08 - 2.77] | 1.01 [0.69 - 1.63] |
|  | T120 | 0.74 [0.61 - 0.88] | 1.61 [1.01 - 2.29] | 1.08 [0.76 - 1.71] |
|  | T180 | 0.65 [0.54 - 0.78] | 1.82 [1.05 - 2.53] | 1.22 [0.90 - 1.57] |
| *Max (pmol/(s*10^6^ PBMCs))* | Baseline | 5.97 [5.12 - 6.06] | 5.89 [5.69 - 6.56] | 6.58 [5.52 - 7.19] |
|  | T060 | 6.93 [6.04 - 7.40] | 6.95 [6.28 - 7.56] | 8.24 [7.03 - 8.66] |
|  | T120 | 6.04 [5.53 - 6.81] | 8.51 [7.90 - 9.79] | 7.71 [7.07 - 8.32] |
|  | T180 | 7.79 [6.82 - 8.25] | 10.64 [7.98 - 12.80] | 9.57 [7.50 - 10.41] |
| *Abbreviations: LPS, lipopolysaccharide; LPS-1, LPS with support initiated at MAP <80mmHg; LPS-2, LPS with support initiated at MAP <65mmHg; Median [Inter Quartile Range]* | | | | |

**Fig. 1:** High-resolution respirometry effect plots; A. Routine, B. Leak, C. Max

*
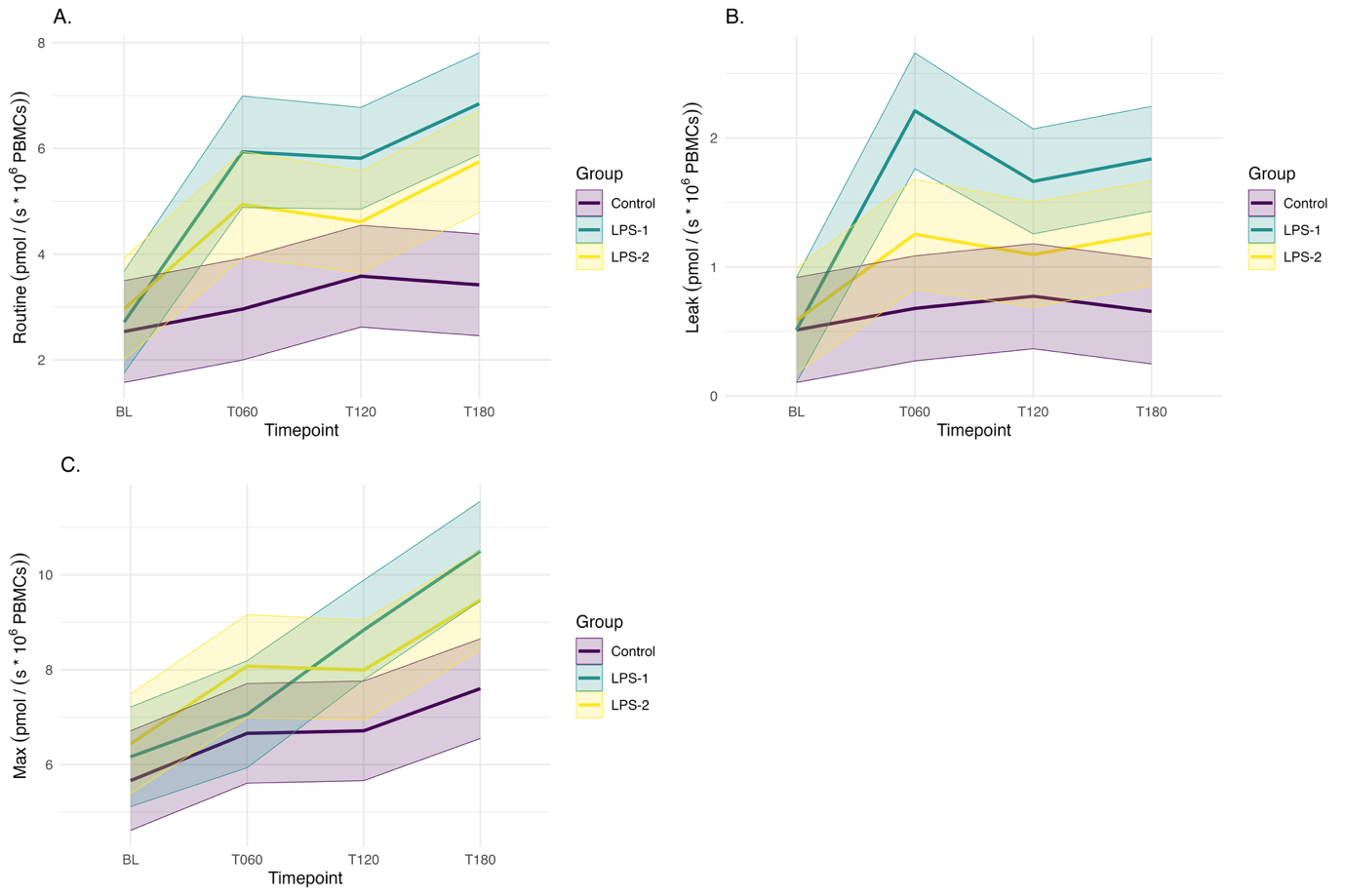
*

*Abbreviations: LPS, lipopolysaccharide; LPS-1, LPS with support initiated at MAP <80mmHg; LPS-2, LPS with support initiated at MAP <65mmHg*

**Table 2:** Linear mixed model outputs for high-resolution respirometry

|  | Routine | | | Leak | | | Max | | |
| --- | --- | --- | --- | --- | --- | --- | --- | --- | --- |
| *Predictors* | *Est.* | *CI* | *P* | *Est.* | *CI* | *P* | *Est.* | *CI* | *P* |
| Control | 2.54 | 1.57 – 3.50 | **<0.001** | 0.51 | 0.10 – 0.92 | **0.014** | 5.66 | 4.61 – 6.71 | **<0.001** |
| Control: T060 | 0.43 | -0.68 – 1.53 | 0.447 | 0.17 | -0.33 – 0.67 | 0.507 | 1.00 | -0.04 – 2.03 | 0.060 |
| Control: T120 | 1.05 | -0.06 – 2.15 | 0.063 | 0.26 | -0.24 – 0.76 | 0.299 | 1.05 | 0.01 – 2.09 | **0.048** |
| Control: T180 | 0.88 | -0.22 – 1.99 | 0.115 | 0.14 | -0.35 – 0.64 | 0.567 | 1.94 | 0.90 – 2.98 | **<0.001** |
| LPS-1 | 0.18 | -1.18 – 1.54 | 0.796 | 0.00 | -0.57 – 0.58 | 0.998 | 0.50 | -0.98 – 1.99 | 0.505 |
| LPS-1: T060 | 2.80 | 1.18 – 4.42 | **0.001** | 1.53 | 0.80 – 2.26 | **<0.001** | -0.10 | -1.62 – 1.42 | 0.898 |
| LPS-1: T120 | 2.05 | 0.49 – 3.61 | **0.011** | 0.89 | 0.19 – 1.59 | **0.014** | 1.63 | 0.16 – 3.09 | **0.030** |
| LPS-1: T180 | 3.25 | 1.68 – 4.81 | **<0.001** | 1.18 | 0.48 – 1.89 | **0.001** | 2.40 | 0.93 – 3.87 | **0.002** |
| LPS-2 | 0.43 | -0.93 – 1.79 | 0.535 | 0.07 | -0.50 – 0.65 | 0.801 | 0.77 | -0.71 – 2.26 | 0.303 |
| LPS-2: T060 | 1.55 | -0.04 – 3.14 | 0.055 | 0.50 | -0.21 – 1.22 | 0.167 | 0.64 | -0.85 – 2.13 | 0.396 |
| LPS-2: T120 | 0.60 | -0.96 – 2.16 | 0.448 | 0.25 | -0.46 – 0.95 | 0.485 | 0.51 | -0.96 – 1.98 | 0.493 |
| LPS-2: T180 | 1.90 | 0.34 – 3.46 | **0.018** | 0.53 | -0.17 – 1.24 | 0.136 | 1.09 | -0.37 – 2.56 | 0.143 |
| *Abbreviations: LPS, lipopolysaccharide; LPS-1, LPS with support initiated at MAP <80mmHg; LPS-2, LPS with support initiated at MAP <65mmHg; Est., estimate; CI, confidence interval* | | | | | | | | | |

2.2 mtDNA and nDNA

Median [IQR] values are presented in Table 3. The mtND1/GCG ratio in PBMCs did not demonstrate significant changes over time in the control group. A significant group-by-time interaction was observed in LPS-1, with higher mtND1/GCG ratios at 120 minutes compared with controls, while a baseline difference was observed in LPS-2 with higher values compared with controls. (Fig. 2A; Table 4).

Plasma mtND1/GCG ratios decreased over time in the control group. A significant group-by-time interaction was observed in LPS-1, with lower values at 60 minutes compared with controls, while LPS-2 showed a different pattern, with persistently lower values and a blunted decline over time. (Fig. 2B; Table 4).

No significant group-by-time interaction or time effect was observed for plasma mtND1 concentrations across groups or time points (Fig. 2C; Table 4).

Plasma GCG concentrations remained stable over time in the control group. A significant group-by-time interaction was observed, with higher plasma GCG concentrations in LPS-1 at 60 and 120 minutes and in LPS-2 at 120 minutes compared with controls (Fig. 2D; Table 4).

**Table 3:** mtDNA and nDNA

|  | Time point | Control (N=10) | LPS-1 (N=10) | LPS-2 (N=10) |
| --- | --- | --- | --- | --- |
| *PBMC mtND1/GCG (ratio)* | Baseline | 105.7 [95.6 - 107.9] | 103.6 [87.0 - 123.2] | 111.4 [105.9 - 122.7] |
|  | T060 | 94.8 [86.6 - 101.2] | 102.1 [96.6 - 115.2] | 113.6 [100.9 - 136.9] |
|  | T120 | 96.4 [91.6 - 101.3] | 112.9 [106.4 - 137.5] | 118.1 [109.5 - 120.9] |
|  | T180 | 94.0 [86.5 - 98.9] | 98.1 [94.7 - 103.1] | 104.2 [98.4 - 111.6] |
| *Plasma mtND1/GCG (ratio)* | Baseline | 595.3 [393.1 - 872.0] | 483.4 [238.3 - 865.7] | 288.0 [253.8 - 319.3] |
|  | T060 | 310.2 [215.1 - 397.8] | 84.8 [39.4 - 138.7] | 46.2 [20.0 - 77.0] |
|  | T120 | 170.1 [111.1 - 221.6] | 18.5 [15.3 - 29.5] | 16.3 [12.3 - 29.4] |
|  | T180 | 96.2 [67.0 - 118.5] | 31.9 [22.7 - 50.9] | 34.2 [24.4 - 50.4] |
| *Plasma mtND1 (copy·µL^-1^)* | Baseline | 151.9 [96.7 - 187.9] | 138.4 [116.5 - 144.1] | 124.0 [69.8 - 153.3] |
|  | T060 | 111.8 [102.7 - 150.2] | 127.5 [105.8 - 152.2] | 93.7 [53.2 - 126.8] |
|  | T120 | 140.0 [79.6 - 176.1] | 99.7 [83.2 - 124.0] | 86.1 [75.1 - 166.4] |
|  | T180 | 154.1 [99.9 - 172.6] | 95.5 [78.0 - 121.4] | 100.3 [58.7 - 153.1] |
| *Plasma GCG (copy·µL^-1^)* | Baseline | 0.20 [0.15 - 0.42] | 0.20 [0.15 - 0.58] | 0.40 [0.36 - 0.50] |
|  | T060 | 0.35 [0.26 - 0.49] | 1.48 [1.13 - 2.60] | 2.20 [1.10 - 3.17] |
|  | T120 | 0.78 [0.60 - 0.93] | 4.83 [3.35 - 7.15] | 4.97 [4.09 - 5.94] |
|  | T180 | 1.57 [0.73 - 2.04] | 2.86 [1.97 - 3.45] | 2.60 [1.65 - 3.85] |
| *Abbreviations: LPS, lipopolysaccharide; LPS-1, LPS with support initiated at MAP <80mmHg; LPS-2, LPS with support initiated at MAP <65mmHg; PBMCs, peripheral blood mononuclear cell; mtND1, Pig NADH Dehydrogenase 1; GCG, Pig Glucagon; Median [Inter Quartile Range]* | | | | |

**Fig. 2:** mtDNA and nDNA effect plots; A. PBMC mtND1/GCG, B. Plasma mtND1/GCG, C. Plasma mtND1, D. Plasma GCG


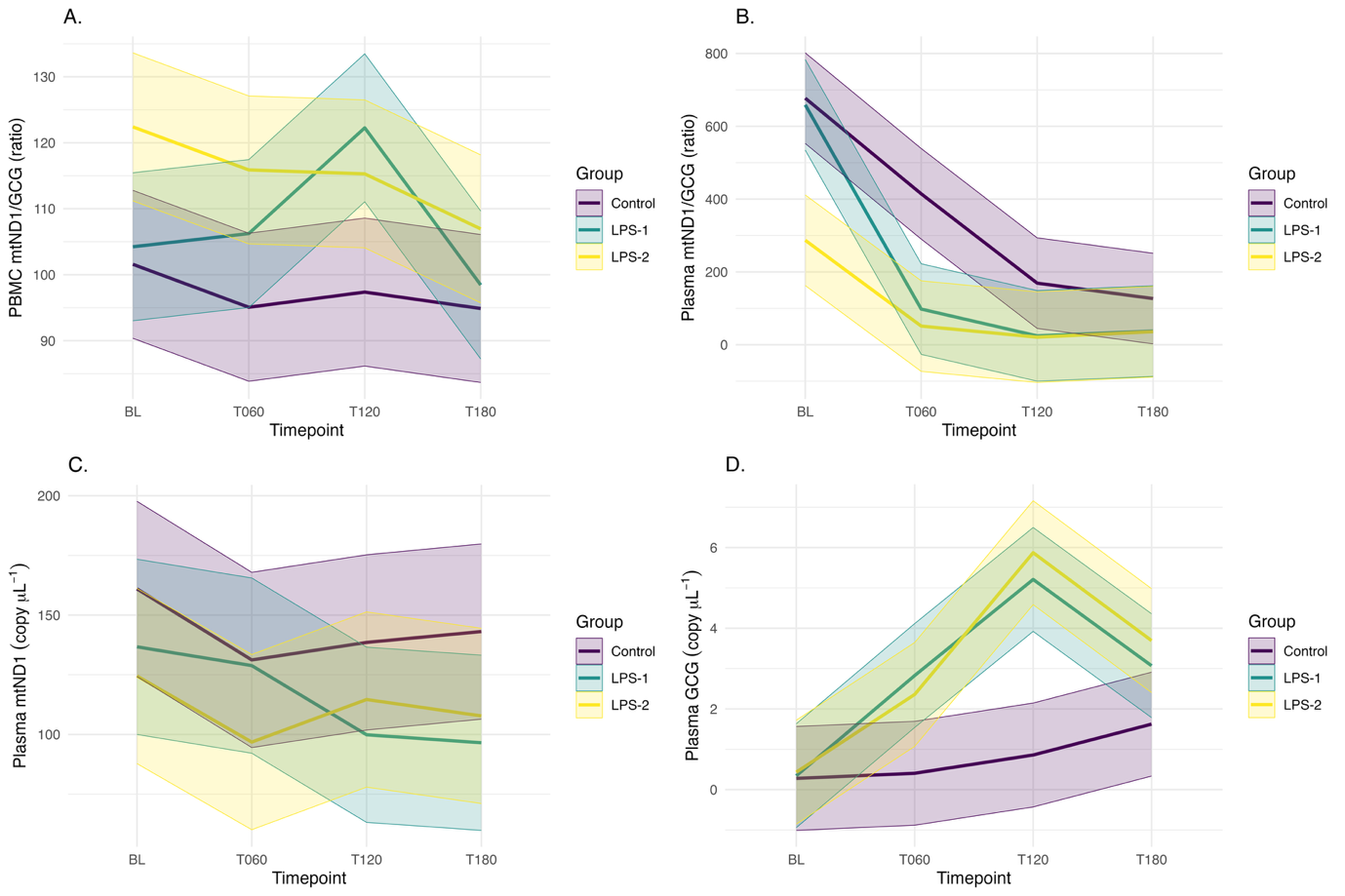


*Abbreviations: LPS, lipopolysaccharide; LPS-1, LPS with support initiated at MAP <80mmHg; LPS-2, LPS with support initiated at MAP <65mmHg; PBMCs, peripheral blood mononuclear cell; mtND1, Pig NADH Dehydrogenase 1; GCG, Pig Glucagon*

**Table 4:** Linear mixed model outputs for mtDNA and nDNA

|  | mtND1/GCG ratio PBMC | | | mtND1/GCG ratio plasma | | | mtND1 plasma | | | GCG plasma | | |
| --- | --- | --- | --- | --- | --- | --- | --- | --- | --- | --- | --- | --- |
| *Predictors* | *Est.* | *CI* | *P* | *Est.* | *CI* | *P* | *Est.* | *CI* | *P* | *Est.* | *CI* | *P* |
| Control | 101.58 | 90.37 – 112.79 | **<0.001** | 677.26 | 552.89 – 801.64 | **<0.001** | 160.96 | 124.26 – 197.67 | **<0.001** | 0.28 | -1.01 – 1.57 | 0.669 |
| Control: T060 | -6.51 | -20.21 – 7.19 | 0.348 | -262.64 | -438.53 – -86.74 | **0.004** | -29.75 | -64.35 – 4.84 | 0.091 | 0.13 | -1.45 – 1.71 | 0.873 |
| Control: T120 | -4.21 | -17.92 – 9.49 | 0.544 | -507.98 | -683.87 – -332.08 | **<0.001** | -22.41 | -57.01 – 12.18 | 0.202 | 0.58 | -1.00 – 2.16 | 0.468 |
| Control: T180 | -6.70 | -20.40 – 7.01 | 0.335 | -550.37 | -726.26 – -374.48 | **<0.001** | -17.87 | -52.46 – 16.73 | 0.308 | 1.35 | -0.23 – 2.92 | 0.094 |
| LPS-1 | 2.66 | -13.20 – 18.51 | 0.741 | -18.25 | -194.15 – 157.64 | 0.837 | -24.26 | -76.17 – 27.66 | 0.356 | 0.07 | -1.75 – 1.89 | 0.943 |
| LPS-1: T060 | 8.51 | -10.87 – 27.89 | 0.386 | -298.33 | -547.09 – -49.58 | **0.019** | 21.88 | -27.04 – 70.81 | 0.377 | 2.36 | 0.12 – 4.59 | **0.039** |
| LPS-1: T120 | 22.25 | 2.86 – 41.63 | **0.025** | -126.54 | -375.29 – 122.21 | 0.315 | -14.43 | -63.35 – 34.50 | 0.560 | 4.28 | 2.05 – 6.52 | **<0.001** |
| LPS-1: T180 | 0.90 | -18.48 – 20.29 | 0.926 | -71.03 | -319.79 – 177.72 | 0.572 | -22.33 | -71.25 – 26.59 | 0.368 | 1.38 | -0.85 – 3.61 | 0.223 |
| LPS-2 | 20.84 | 4.98 – 36.69 | **0.011** | -390.23 | -566.12 – -214.33 | **<0.001** | -36.47 | -88.38 – 15.44 | 0.167 | 0.15 | -1.67 – 1.98 | 0.866 |
| LPS-2: T060 | -0.02 | -19.40 – 19.36 | 0.998 | 26.89 | -221.86 – 275.64 | 0.831 | 2.06 | -46.86 – 50.98 | 0.934 | 1.80 | -0.43 – 4.03 | 0.113 |
| LPS-2: T120 | -2.91 | -22.30 – 16.47 | 0.766 | 241.75 | -7.01 – 490.50 | 0.057 | 12.56 | -36.36 – 61.48 | 0.612 | 4.86 | 2.63 – 7.09 | **<0.001** |
| LPS-2: T180 | -8.77 | -28.15 – 10.61 | 0.372 | 299.25 | 50.50 – 548.00 | **0.019** | 1.12 | -47.80 – 50.04 | 0.964 | 1.91 | -0.32 – 4.15 | 0.092 |
| *Abbreviations:* PBMCs, peripheral blood mononuclear cell; mtND1, Pig NADH Dehydrogenase 1; GCG, Pig Glucagon; *LPS, lipopolysaccharide; LPS-1, LPS with support initiated at MAP <80mmHg; LPS-2, LPS with support initiated at MAP <65mmHg; Est., estimate; CI, confidence interval* | | | | | | | | | | | | |

2.3 Histopathology

Across all groups and organs, the proportion of HIF-1α–positive cells remained low (<1.2%) and did not differ between groups (Table 5).

**Table 5:** HIF-1α immunostaining mean ± SD

|  | **Control (N=9)** | **LPS-1 (N=8)** | **LPS-2 (N=9)** |
| --- | --- | --- | --- |
| **Kidney (% positive)** | | | |
| T180 | 0.09 ± 0.07 | 0.30 ± 0.25 | 0.34 ± 0.25 |
| **Liver (% positive)** | | | |
| T180 | 1.07 ± 1.03 | 0.74 ± 1.16 | 1.08 ± 2.26 |
| **Intestine (% positive)** | | | |
| T180 | 0.71 ± 0.73 | 0.40 ± 0.38 | 0.29 ± 0.24 |
| *Abbreviations: LPS, lipopolysaccharide; LPS with support initiated at MAP <80mmHg; LPS-2, LPS with support initiated at MAP <65mmHg* | | | |

**3. Discussion**

During early endotoxemia, PBMC mitochondrial respiration demonstrated dynamic changes, with increased routine and maximal respiration, particularly in LPS-1 and to a lesser extent in LPS-2. These findings suggest increased mitochondrial respiratory flux under endotoxemic conditions. However, routine respiration reflects both ATP-linked and non–ATP-linked components and therefore cannot be interpreted as a direct surrogate for ATP production [4].

Leak respiration was predominantly increased in LPS-1, suggesting altered mitochondrial coupling efficiency. This may reflect adaptive or stress-related changes in mitochondrial function, including increased proton leak, ion transport, or redox signaling, rather than unequivocal mitochondrial dysfunction. Maximal respiration increased over time in the control group and was higher in LPS-1 at later time points, indicating preserved or even enhanced mitochondrial respiratory capacity during the early phase of endotoxemia. These findings are consistent with observations summarized by Singer et al., describing an early hyperdynamic mitochondrial response following endotoxin exposure [5]. In contrast, reduced mitochondrial respiration at later stages has been reported by Kahoutová et al. in a swine sepsis model, likely reflecting time-dependent progression toward mitochondrial dysfunction [6].

In parallel, PBMC mtDNA-to-nDNA ratios remained largely unchanged, whereas plasma mtDNA-to-nDNA ratios decreased over time across groups. This reduction appeared primarily driven by increasing plasma nDNA concentrations, likely reflecting surgical tissue injury. The absence of elevated circulating mtDNA in the LPS groups may relate to the early timing of measurements, as increased plasma mtDNA has been reported later in sepsis and associated with disease severity and mortality [7, 8].

Importantly, these findings highlight a divergence between ex vivo PBMC mitochondrial respiration and in vivo tissue mitoVO_2_ during early endotoxemia. While PBMCs exhibited increased respiratory activity *ex vivo*, tissue-level mitoVO_2_ was reduced or blunted in selected organs *in vivo*. This discrepancy may reflect the influence of local factors within the intact physiological environment, including microvascular alterations, inflammatory signaling, and substrate availability, which are not captured in *ex vivo* measurements. Taken together, these observations support the concept that mitochondrial responses during endotoxemia are highly time- and context-dependent.

**References:**

1. Smolkova K, Bellance N, Scandurra F, Genot E, Gnaiger E, Plecita-Hlavata L, et al. Mitochondrial bioenergetic adaptations of breast cancer cells to aglycemia and hypoxia. J Bioenerg Biomembr. 2010;42(1):55-67.

2. Gnaiger E, Steinlechner-Maran R, Mendez G, Eberl T, Margreiter R. Control of mitochondrial and cellular respiration by oxygen. J Bioenerg Biomembr. 1995;27(6):583-96.

3. Streng L, de Wijs CJ, Raat NJH, Specht PAC, Sneiders D, van der Kaaij M, et al. In Vivo and Ex Vivo Mitochondrial Function in COVID-19 Patients on the Intensive Care Unit. Biomedicines. 2022;10(7).

4. Gnaiger E. Mitochondrial pathways and respiratory control : an introduction to OXPHOS analysis. Innsbruck: OROBOROS Instruments GmbH; 2020.

5. Singer M. Mitochondrial function in sepsis: acute phase versus multiple organ failure. Crit Care Med. 2007;35(9 Suppl):S441-8.

6. Kohoutova M, Horak J, Jarkovska D, Martinkova V, Tegl V, Nalos L, et al. Vagus Nerve Stimulation Attenuates Multiple Organ Dysfunction in Resuscitated Porcine Progressive Sepsis. Crit Care Med. 2019;47(6):e461-e9.

7. West AP, Shadel GS. Mitochondrial DNA in innate immune responses and inflammatory pathology. Nat Rev Immunol. 2017;17(6):363-75.

8. Krychtiuk KA, Ruhittel S, Hohensinner PJ, Koller L, Kaun C, Lenz M, et al. Mitochondrial DNA and Toll-Like Receptor-9 Are Associated With Mortality in Critically Ill Patients. Crit Care Med. 2015;43(12):2633-41.
