# Supplementary material for: In vivo mitochondrial oxygen consumption during LPS-induced endotoxemia: a controlled experimental study in swine
Source: Intensive Care Med Exp. 2026 Jul 20;14:97. doi: 10.1186/s40635-026-00951-z (PMC13385532; doi:10.1186/s40635-026-00951-z)
Supplement: Supplementary file 3 — Supplementary Material 3 [file 40635_2026_951_MOESM3_ESM.docx]

**Additional file 3:** Organ function and injury markers

**1. Methods**

Liver function was assessed using indocyanine green (ICG) clearance expressed as the ICG plasma disappearance rate (ICG-PDR) in % per minute. ICG is a dye that binds to plasma proteins and is exclusively excreted by the liver without undergoing enterohepatic circulation, making it suitable for hepatic function evaluation [1, 2].

For each ICG clearance measurement, ICG (0.5 mg•kg^-1^) (Carl Roth GmbH & Co. KG, Karlsruhe, Germany) was dissolved in sterile water until a concentration of 2.5 mg•mL^-1^ was reached. This solution was administered at t=0 of each ICG-clearance measurement through the right jugular vein in 5 seconds. At t=1, 2, 3, 5, 7, 10, 12 minutes a 1 mL blood sample was collected in a heparinized syringe. Immediately after the blood withdrawal, samples were centrifuged at 7000 rpm for 10 minutes. 200 μL of each sample was pipetted into a 96-well plate. The ICG concentration was spectrophotometrically measured at 805 nm using an ELISA reader (VersaMax; Molecular Devices, Sunnyvale, CA). ICG measurements were initiated 15 minutes prior to the baseline and repeated at each subsequent time point. ICG-clearance was expressed as plasma disappearance rate (PDR-ICG), in % per minute.

Kidney function was evaluated by creatinine clearance, calculated for the right kidney only. Creatinine clearance was calculated as an index of glomerular filtration rate using the standard formula clearance (mL•min^-1^) = (U × V)/P, where U represents urine creatinine concentration, V represents urine volume per time, and P represents plasma creatinine concentration.

Urine samples were collected from the right ureter catheter at 10-minute intervals and centrifuged at 7000 rpm for 10 minutes at room temperature to determine urine volume and concentration. Arterial blood samples were collected at the same time in BD Vacutainer^®^ Barricor™ Plasma blood collection tubes and centrifuged at 4000× g for 3 minutes at room temperature. Creatinine concentrations in both urine and plasma were measured using colorimetric methods.

Urinary neutrophil gelatinase-associated lipocalin (NGAL) concentration was measured using the Pig Lipocalin-2 ELISA Kit (ab207924, Abcam, Cambridge, UK). Plasma intestinal fatty acid binding protein (I-FABP) concentration was measured using the Pig FABP2 (Intestinal Fatty Acid Binding Protein) ELISA Kit (MBS8807266, MyBiosource, San Diego, USA).

**2. Results**

Median [IQR] values are presented in Table 1. Right kidney clearance did not demonstrate a significant time effect in the control or LPS-1 (LPS with hemodynamic support initiated at MAP <80 mmHg). A significant group-by-time interaction was observed in LPS-2 (LPS with hemodynamic support initiated at MAP <65 mmHg), with lower values at 120 minutes compared with controls (**Fig.** 1A; Table 2).

NGAL demonstrated a significant group-by-time interaction. NGAL concentrations were higher in LPS-2 at 180 minutes, with a smaller increase also observed in LPS-1 at the same time point (**Fig.** 1B; Table 2).

ICG-PDR decreased over time in the control group. A significant group-by-time interaction was observed in both LPS groups, with further reductions compared with controls at 60, 120, and 180 minutes in LPS-1, and at 120 and 180 minutes in LPS-2 (**Fig.** 1C; Table 2). ICG-PDR was inversely correlated with arterial lactate concentrations (**Fig.** 2).

I-FABP increased over time in the control group. No significant group-by-time interaction was observed in either LPS group (**Fig.** 1D; Table 2).

**Table 1:** Organ function and injury markers

|  | Time point | Control (N=10) | LPS-1 (N=10) | LPS-2 (N=10) |
| --- | --- | --- | --- | --- |
| *Right kidney clearance (ml·min^-1^)* | Baseline | 41.7 [38.2 - 49.7] | 44.0 [41.6 - 47.8] | 37.8 [34.3 - 45.8] |
|  | T060 | 43.7 [34.8 - 46.9] | 42.6 [39.9 - 49.2] | 36.7 [31.5 - 64.0] |
|  | T120 | 48.5 [41.7 - 57.8] | 37.2 [32.4 - 45.1] | 28.7 [24.8 - 31.6] |
|  | T180 | 45.8 [40.5 - 48.9] | 41.1 [36.3 - 49.3] | 29.9 [25.5 - 36.8] |
| *NGAL (ng·mL^-1^)* | Baseline | 5.57 [4.28 - 6.55] | 5.56 [3.18 - 7.74] | 4.27 [3.00 - 4.88] |
|  | T060 | 5.43 [3.81 - 7.83] | 4.76 [3.90 - 11.37] | 11.64 [7.81 - 13.20] |
|  | T120 | 5.19 [4.62 - 7.61] | 6.62 [4.92 - 9.22] | 13.53 [9.55 - 20.11] |
|  | T180 | 5.42 [3.85 - 6.92] | 11.55 [10.22 - 19.16] | 36.49 [16.90 - 65.22] |
| *ICG-PDR (%·min^-1^)* | Baseline | 13.43 [11.82 - 14.94] | 14.15 [12.85 - 15.32] | 11.20 [11.11 - 12.91] |
|  | T060 | 12.15 [10.78 - 13.22] | 8.94 [8.61 - 11.42] | 9.47 [7.65 - 10.99] |
|  | T120 | 10.00 [8.18 - 10.35] | 4.85 [4.40 - 5.89] | 4.91 [3.80 - 5.71] |
|  | T180 | 6.94 [6.34 - 7.19] | 3.85 [2.89 - 4.52] | 3.99 [3.26 - 4.92] |
| *I-FABP (pg·mL^-1^)* | Baseline | 65.4 [52.4 - 76.4] | 56.5 [44.5 - 79.4] | 76.8 [59.8 - 94.6] |
|  | T060 | 74.2 [56.6 - 91.5] | 68.7 [50.7 - 88.6] | 83.2 [65.9 - 95.7] |
|  | T120 | 66.4 [55.1 - 105.6] | 68.6 [56.8 - 75.4] | 83.5 [70.4 - 96.1] |
|  | T180 | 81.9 [67.6 - 111.7] | 71.0 [63.1 - 75.9] | 76.9 [70.4 - 106.3] |
| *Abbreviations: LPS, lipopolysaccharide; LPS-1, LPS with support initiated at MAP <80mmHg; LPS-2, LPS with support initiated at MAP <65mmHg; ICG-PDR, indocyanine green plasma disappearance rate; NGAL, Neutrophil gelatinase-associated lipocalin; I-FABP, intestinal fatty acid binding protein; Median [Inter Quartile Range]* | | | | |

**Fig. 1:** Organ function and injury markers effect plots; A. Right kidney clearance, B. NGAL, C. ICG-PDR, D. I-FABP


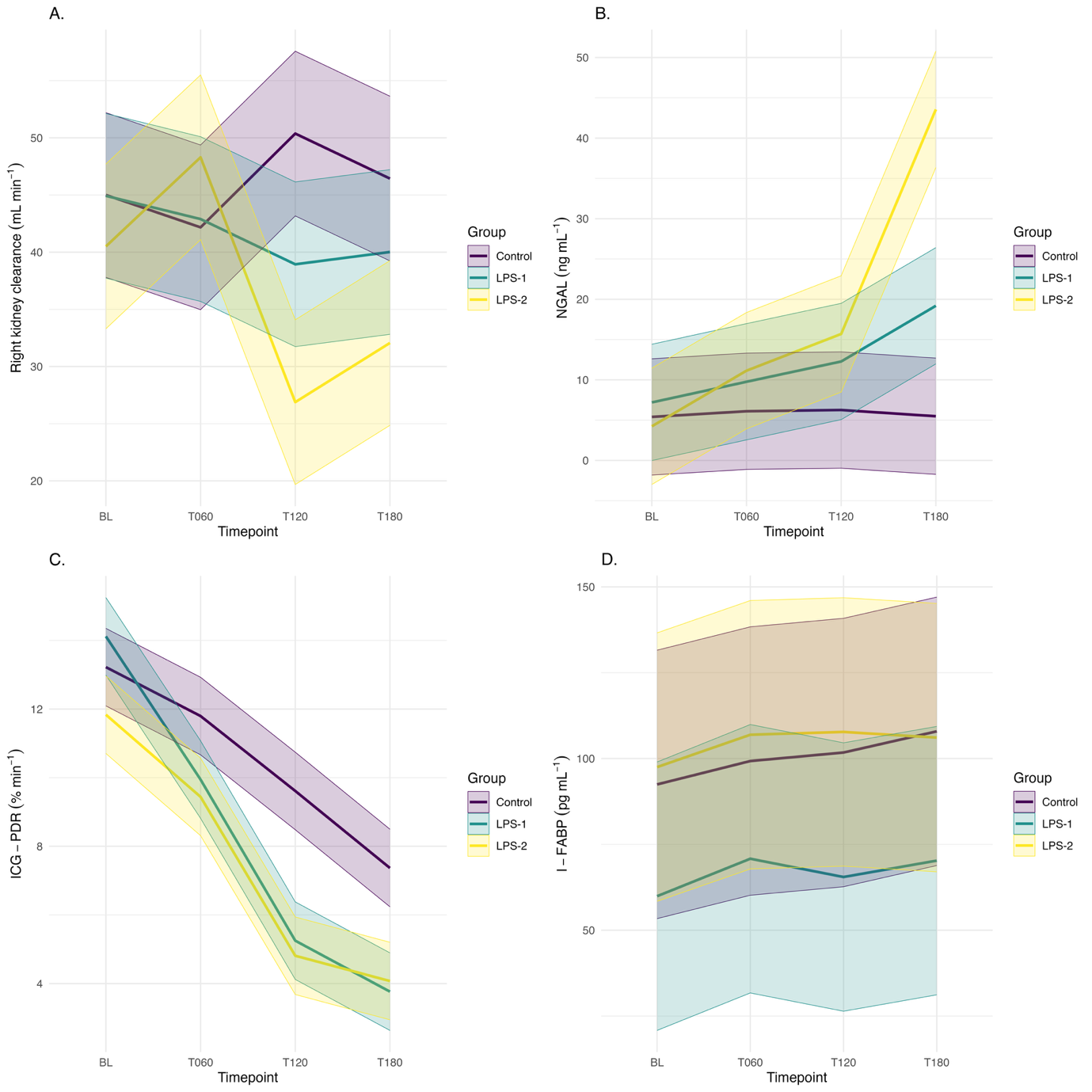


*Abbreviations: LPS, lipopolysaccharide; LPS-1, LPS with support initiated at MAP <80mmHg; LPS-2, LPS with support initiated at MAP <65mmHg; ICG-PDR, indocyanine green plasma disappearance rate; NGAL, Neutrophil gelatinase-associated lipocalin; I-FABP, intestinal fatty acid binding protein*

**Table 2:** Linear mixed model outputs for organ function and injury markers

|  | Clearance right kidney | | | NGAL | | | ICG-PDR | | | I-FABP | | |
| --- | --- | --- | --- | --- | --- | --- | --- | --- | --- | --- | --- | --- |
| *Predictors* | *Est.* | *CI* | *P* | *Est.* | *CI* | *P* | *Est.* | *CI* | *P* | *Est.* | *CI* | *P* |
| Control | 45.00 | 37.80 – 52.20 | **<0.001** | 5.40 | -1.81 – 12.62 | 0.141 | 13.22 | 12.09 – 14.35 | **<0.001** | 92.46 | 53.38 – 131.55 | **<0.001** |
| Control: T060 | -2.83 | -11.67 – 6.01 | 0.527 | 0.70 | -7.31 – 8.72 | 0.862 | -1.43 | -2.54 – -0.32 | **0.012** | 6.80 | -1.71 – 15.32 | 0.116 |
| Control: T120 | 5.37 | -3.47 – 14.21 | 0.231 | 0.85 | -7.16 – 8.87 | 0.833 | -3.61 | -4.72 – -2.50 | **<0.001** | 9.28 | 0.77 – 17.80 | **0.033** |
| Control: T180 | 1.43 | -7.41 – 10.28 | 0.748 | 0.08 | -7.93 – 8.10 | 0.983 | -5.85 | -6.97 – -4.74 | **<0.001** | 15.45 | 6.94 – 23.97 | **<0.001** |
| LPS-1 | -0.07 | -10.26 – 10.11 | 0.989 | 1.80 | -8.41 – 12.00 | 0.728 | 0.90 | -0.70 – 2.49 | 0.268 | -32.55 | -87.82 – 22.72 | 0.246 |
| LPS-1: T060 | 0.80 | -11.71 – 13.30 | 0.900 | 1.86 | -9.47 – 13.19 | 0.745 | -2.75 | -4.32 – -1.18 | **0.001** | 4.11 | -7.93 – 16.15 | 0.500 |
| LPS-1: T120 | -11.36 | -23.86 – 1.15 | 0.075 | 4.23 | -7.11 – 15.56 | 0.461 | -5.26 | -6.83 – -3.69 | **<0.001** | -3.70 | -15.74 – 8.34 | 0.543 |
| LPS-1: T180 | -6.35 | -18.85 – 6.16 | 0.317 | 11.91 | 0.57 – 23.24 | **0.040** | -4.50 | -6.07 – -2.93 | **<0.001** | -5.09 | -17.13 – 6.95 | 0.404 |
| LPS-2 | -4.50 | -14.68 – 5.69 | 0.383 | -1.17 | -11.38 – 9.03 | 0.820 | -1.39 | -2.98 – 0.21 | 0.088 | 5.05 | -50.23 – 60.32 | 0.857 |
| LPS-2: T060 | 10.62 | -1.88 – 23.13 | 0.095 | 6.20 | -5.13 – 17.54 | 0.280 | -0.97 | -2.54 – 0.60 | 0.224 | 2.61 | -9.43 – 14.65 | 0.668 |
| LPS-2: T120 | -18.99 | -31.50 – -6.48 | **0.003** | 10.61 | -0.72 – 21.94 | 0.066 | -3.42 | -4.99 – -1.85 | **<0.001** | 0.96 | -11.08 – 13.00 | 0.875 |
| LPS-2: T180 | -9.88 | -22.38 – 2.63 | 0.120 | 39.25 | 27.92 – 50.58 | **<0.001** | -1.91 | -3.48 – -0.34 | **0.018** | -6.87 | -18.91 – 5.17 | 0.261 |
| *Abbreviations: ICG-PDR, indocyanine green plasma disappearance rate; NGAL, Neutrophil gelatinase-associated lipocalin; I-FABP, intestinal fatty acid binding protein; LPS, lipopolysaccharide; LPS-1, LPS with support initiated at MAP <80mmHg; LPS-2, LPS with support initiated at MAP <65mmHg; Est., estimate; CI, confidence interval* | | | | | | | | | | | | |

**Fig. 2:** Correlation plot of lactate and ICG-PDR


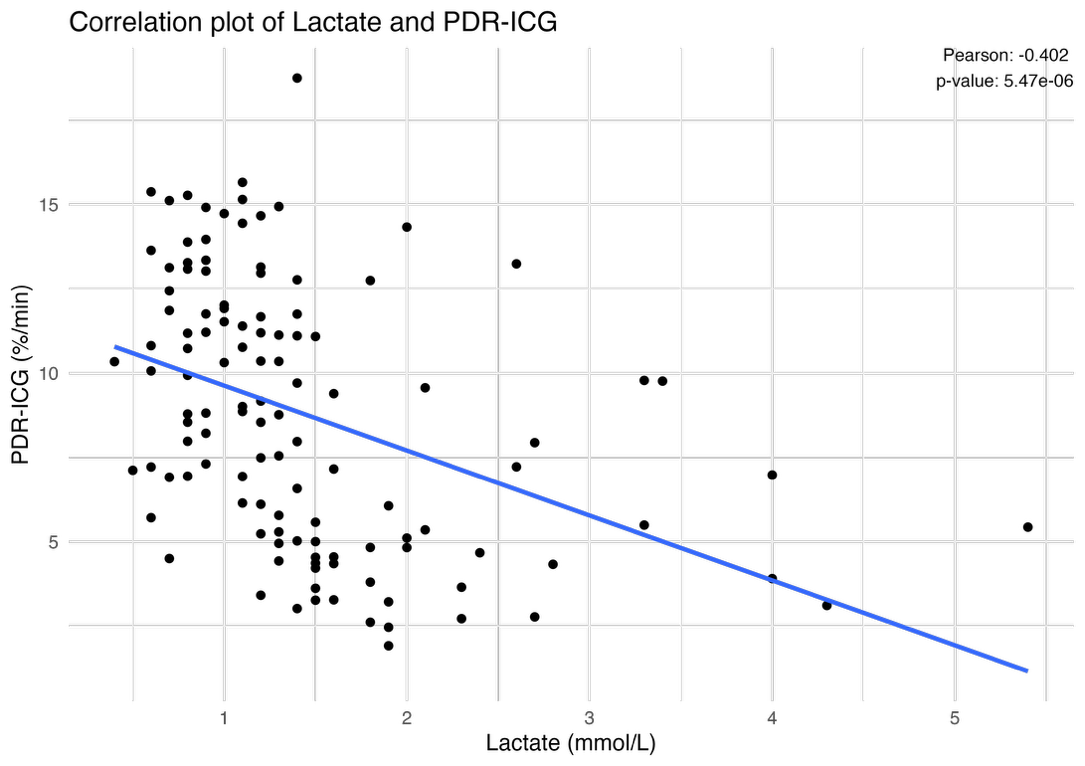


*Abbreviations: ICG-PDR, indocyanine green plasma disappearance rate*

**3. Discussion**

A downward trend in ICG-PDR, indicative of reduced hepatic clearance capacity [3], was observed across all groups, with a more pronounced decline in the LPS-treated animals. The reduction in the control group likely reflects the physiological impact of anesthesia and major surgery, whereas endotoxemia appeared to exacerbate this decline in both LPS groups. In parallel, arterial lactate concentrations increased, particularly in the LPS groups. Although hyperlactatemia is often interpreted as a marker of anaerobic metabolism, the significant negative correlation between ICG-PDR and lactate suggests that impaired hepatic clearance may have contributed to lactate accumulation, rather than lactate production being driven solely driven by tissue hypoxia. This interpretation is supported by the absence of increased HIF-1α immunostaining in hepatic tissue, indicating no histological evidence of cellular hypoxia during the observation period [4].

At the mitochondrial level, liver mitoPO_2_ remained largely preserved in LPS-1, while liver mitoVO_2_ was lower at later time points compared with controls, reflecting a blunted increase rather than an absolute decline. Together, these findings suggest that hepatic mitochondrial oxygen utilization may be altered during early endotoxemia even in the absence of overt hypoperfusion or cellular hypoxia. This is consistent with prior observations by Tapia et al., who reported impaired lactate clearance during endotoxic shock despite preserved hepatic blood flow [5], and by Bakker et al., who emphasized the importance of distinguishing impaired lactate clearance from increased lactate production when interpreting hyperlactatemia in critically ill patients [6]. Collectively, these data support the interpretation that lactate elevation during early endotoxemia reflects both metabolic responses and reduced hepatic clearance capacity.

Renal function exhibited a distinct and time-dependent pattern. Right kidney clearance was lower in LPS-2 at 120 minutes, coinciding with a greater hypotension burden as reflected by increased time-weighted average MAP <60 mmHg. This association is consistent with clinical observations linking cumulative hypotension exposure to acute kidney injury, as reported by Maheshwari et al., although causality cannot be inferred in the present model [7]. At 180 minutes, both LPS groups demonstrated increased urinary NGAL concentrations, with substantially higher levels in LPS-2, suggesting a greater degree of renal injury under more permissive hypotension [8].

These renal injury signals occurred alongside transient increases in renal cortex mitoPO_2_ and mitoVO_2_ early after LPS administration, particularly in LPS-2. This pattern may reflect regional heterogeneity in renal oxygenation rather than improved tissue oxygen delivery. Experimental and computational models have highlighted the vulnerability of the renal medulla to hypoxia due to its high metabolic demand, relatively low blood flow, and susceptibility to oxygen shunting from the cortex [9]. Thus, elevated cortical oxygenation does not preclude concurrent medullary hypoxia, which may contribute to early renal injury despite preserved or increased cortical mitoPO_2_ [10]. These findings highlight the complexity of renal oxygen handling during endotoxemia and caution against interpreting cortical oxygenation measurements as representative of whole-organ oxygen balance.

**References:**

1. De Gasperi A, Mazza E, Prosperi M. Indocyanine green kinetics to assess liver function: Ready for a clinical dynamic assessment in major liver surgery? World J Hepatol. 2016;8(7):355-67.

2. Leevy CM, Smith F, Longueville J, Paumgartner G, Howard MM. Indocyanine green clearance as a test for hepatic function. Evaluation by dichromatic ear densitometry. JAMA. 1967;200(3):236-40.

3. Sakka SG. Assessing liver function. Curr Opin Crit Care. 2007;13(2):207-14.

4. Weidemann A, Johnson RS. Biology of HIF-1alpha. Cell Death Differ. 2008;15(4):621-7.

5. Tapia P, Soto D, Bruhn A, Alegria L, Jarufe N, Luengo C, et al. Impairment of exogenous lactate clearance in experimental hyperdynamic septic shock is not related to total liver hypoperfusion. Crit Care. 2015;19(1):188.

6. Bakker J, de Backer D, Hernandez G. Lactate-guided resuscitation saves lives: we are not sure. Intensive Care Med. 2016;42(3):472-4.

7. Maheshwari K, Nathanson BH, Munson SH, Khangulov V, Stevens M, Badani H, et al. The relationship between ICU hypotension and in-hospital mortality and morbidity in septic patients. Intensive Care Med. 2018;44(6):857-67.

8. Soni SS, Cruz D, Bobek I, Chionh CY, Nalesso F, Lentini P, et al. NGAL: a biomarker of acute kidney injury and other systemic conditions. Int Urol Nephrol. 2010;42(1):141-50.

9. Lee CJ, Gardiner BS, Evans RG, Smith DW. A model of oxygen transport in the rat renal medulla. Am J Physiol Renal Physiol. 2018;315(6):F1787-F811.

10. Brezis M, Rosen S. Hypoxia of the renal medulla--its implications for disease. N Engl J Med. 1995;332(10):647-55.
