# Supplementary figures and images for: In vivo mitochondrial oxygen consumption during LPS-induced endotoxemia: a controlled experimental study in swine
Source: Intensive Care Med Exp. 2026 Jul 20;14:97. doi: 10.1186/s40635-026-00951-z (PMC13385532; doi:10.1186/s40635-026-00951-z)

**Additional file 4:** Time-weighted averages of MAP


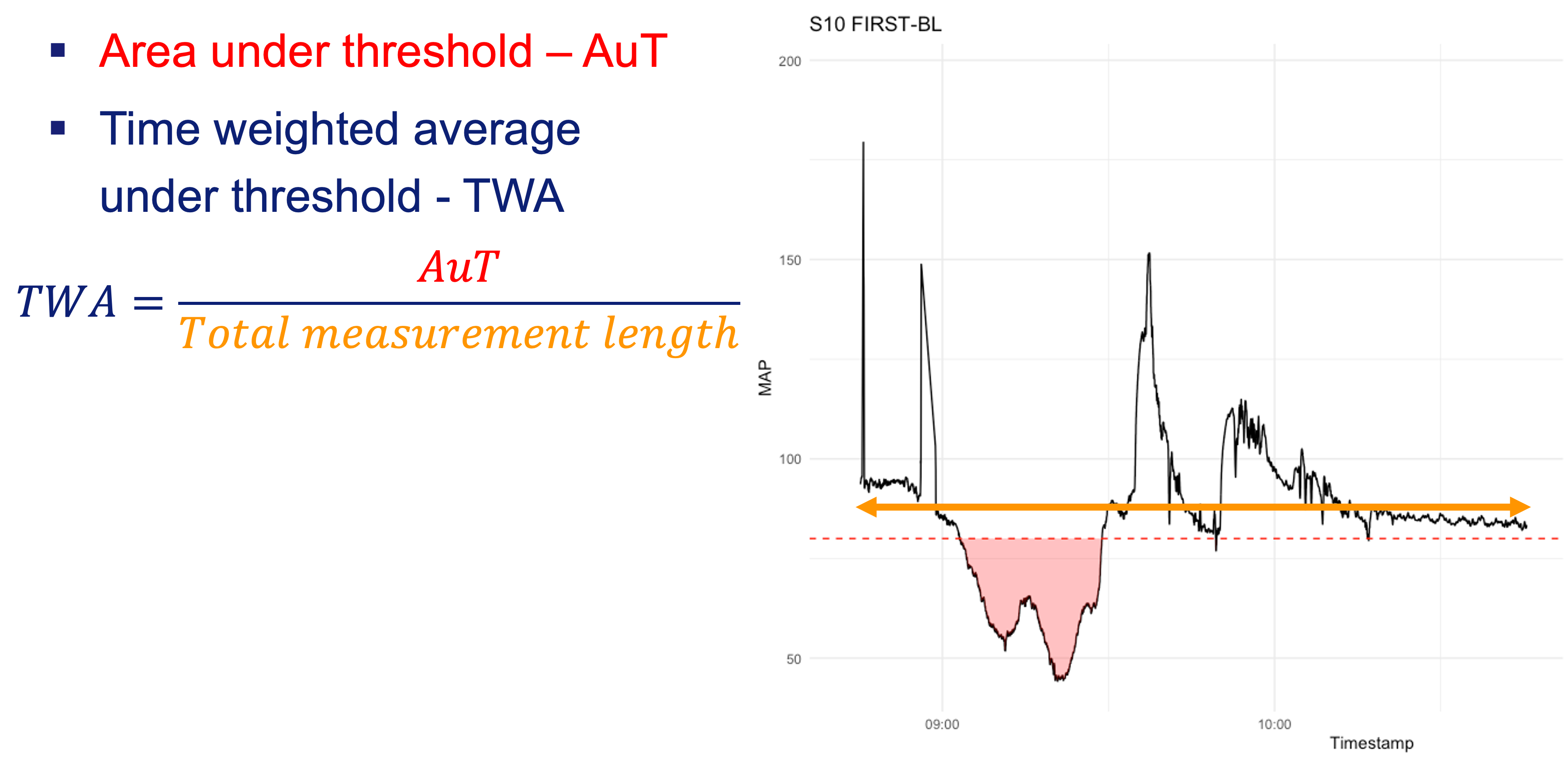

Supplement: Supplementary file 4 — Supplementary Material 4 [file 40635_2026_951_MOESM4_ESM.docx]
