# Supplementary material for: In vivo mitochondrial oxygen consumption during LPS-induced endotoxemia: a controlled experimental study in swine
Source: Intensive Care Med Exp. 2026 Jul 20;14:97. doi: 10.1186/s40635-026-00951-z (PMC13385532; doi:10.1186/s40635-026-00951-z)
Supplement: Supplementary file 5 — Supplementary Material 5 [file 40635_2026_951_MOESM5_ESM.docx]

**Additional file 5:** Linear mixed model outputs

**Table 1:** Linear mixed model outputs for heartrate, MAP, TWA-MAP <60 mmHg and <80 mmHg.

|  | Heartrate | | | MAP | | | TWA-MAP <60 mmHg | | | TWA-MAP <80 mmHg | | |
| --- | --- | --- | --- | --- | --- | --- | --- | --- | --- | --- | --- | --- |
| *Predictors* | *Est.* | *CI* | *P* | *Est.* | *CI* | *P* | *Est.* | *CI* | *P* | *Est.* | *CI* | *P* |
| Control | 94.80 | 84.20 – 105.40 | **<0.001** | 86.00 | 80.11 – 91.89 | **<0.001** | 0.17 | -0.35 – 0.70 | 0.511 | 1.70 | -0.57 – 3.97 | 0.140 |
| Control: T060 | 19.70 | 7.77 – 31.63 | **0.001** | -1.00 | -7.39 – 5.39 | 0.757 | -0.01 | -0.65 – 0.63 | 0.970 | 1.25 | -1.05 – 3.54 | 0.284 |
| Control: T120 | 31.00 | 19.07 – 42.93 | **<0.001** | 2.00 | -4.39 – 8.39 | 0.536 | -0.01 | -0.65 – 0.63 | 0.972 | 0.47 | -1.83 – 2.76 | 0.687 |
| Control: T180 | 35.50 | 23.57 – 47.43 | **<0.001** | 8.40 | 2.01 – 14.79 | **0.010** | 0.00 | -0.64 – 0.64 | 0.993 | -0.23 | -2.53 – 2.06 | 0.841 |
| LPS-1 | 4.10 | -10.88 – 19.08 | 0.589 | -1.80 | -10.13 – 6.53 | 0.669 | -0.02 | -0.75 – 0.72 | 0.963 | 0.06 | -3.14 – 3.27 | 0.969 |
| LPS-1: T060 | 19.80 | 2.93 – 36.67 | **0.022** | -5.20 | -14.24 – 3.84 | 0.256 | 0.03 | -0.87 – 0.94 | 0.940 | -0.18 | -3.42 – 3.06 | 0.913 |
| LPS-1: T120 | 36.00 | 19.13 – 52.87 | **<0.001** | -19.10 | -28.14 – -10.06 | **<0.001** | 0.22 | -0.68 – 1.12 | 0.627 | 8.70 | 5.45 – 11.94 | **<0.001** |
| LPS-1: T180 | 18.80 | 1.93 – 35.67 | **0.029** | -29.40 | -38.44 – -20.36 | **<0.001** | 2.45 | 1.55 – 3.35 | **<0.001** | 14.54 | 11.29 – 17.78 | **<0.001** |
| LPS-2 | 11.70 | -3.28 – 26.68 | 0.125 | 0.80 | -7.53 – 9.13 | 0.849 | 0.32 | -0.42 – 1.06 | 0.395 | 1.40 | -1.81 – 4.61 | 0.388 |
| LPS-2: T060 | 19.00 | 2.13 – 35.87 | **0.028** | -11.30 | -20.34 – -2.26 | **0.015** | 0.45 | -0.46 – 1.35 | 0.328 | 0.89 | -2.36 – 4.13 | 0.589 |
| LPS-2: T120 | 35.40 | 18.53 – 52.27 | **<0.001** | -24.90 | -33.94 – -15.86 | **<0.001** | 1.70 | 0.79 – 2.60 | **<0.001** | 12.13 | 8.88 – 15.37 | **<0.001** |
| LPS-2: T180 | 34.70 | 17.83 – 51.57 | **<0.001** | -16.80 | -25.84 – -7.76 | **<0.001** | 0.29 | -0.62 – 1.19 | 0.531 | 10.16 | 6.92 – 13.40 | **<0.001** |
| *Abbreviations: MAP, mean arterial pressure; TWA-MAP, time weighted average mean arterial pressure; LPS, lipopolysaccharide; LPS-1, LPS with support initiated at MAP <80mmHg; LPS-2, LPS with support initiated at MAP <65mmHg; Est., estimate; CI, confidence interval* | | | | | | | | | | | | |

**Table 2:** Linear mixed model outputs for lactate and temperature

|  | Lactate | | | Temperature | | |
| --- | --- | --- | --- | --- | --- | --- |
| *Predictors* | *Est.* | *CI* | *P* | *Est.* | *CI* | *P* |
| Control | 1.09 | 0.71 – 1.47 | **<0.001** | 38.58 | 38.28 – 38.88 | **<0.001** |
| Control: T060 | -0.14 | -0.54 – 0.26 | 0.492 | 0.75 | 0.45 – 1.05 | **<0.001** |
| Control: T120 | -0.27 | -0.67 – 0.13 | 0.187 | 0.86 | 0.56 – 1.16 | **<0.001** |
| Control: T180 | -0.34 | -0.74 – 0.06 | 0.097 | 0.82 | 0.52 – 1.12 | **<0.001** |
| LPS-1 | 0.02 | -0.52 – 0.56 | 0.942 | 0.28 | -0.14 – 0.70 | 0.193 |
| LPS-1: T060 | 0.19 | -0.38 – 0.76 | 0.510 | 0.12 | -0.31 – 0.55 | 0.581 |
| LPS-1: T120 | 0.65 | 0.08 – 1.22 | **0.026** | -0.11 | -0.54 – 0.32 | 0.613 |
| LPS-1: T180 | 1.23 | 0.66 – 1.80 | **<0.001** | -0.47 | -0.90 – -0.04 | **0.033** |
| LPS-2 | 0.30 | -0.24 – 0.84 | 0.274 | 0.02 | -0.40 – 0.44 | 0.926 |
| LPS-2: T060 | 0.77 | 0.20 – 1.34 | **0.009** | 0.42 | -0.01 – 0.85 | 0.056 |
| LPS-2: T120 | 0.96 | 0.39 – 1.53 | **0.001** | 0.40 | -0.03 – 0.83 | 0.068 |
| LPS-2: T180 | 1.45 | 0.88 – 2.02 | **<0.001** | 0.33 | -0.10 – 0.76 | 0.131 |
| *Abbreviations: LPS, lipopolysaccharide; LPS-1, LPS with support initiated at MAP <80mmHg; LPS-2, LPS with support initiated at MAP <65mmHg; Est., estimate; CI, confidence interval* | | | | | | |

**Table 3:** Linear mixed model outputs for infusion rate and noradrenaline

|  | Infusion rate | | | Noradrenaline | | |
| --- | --- | --- | --- | --- | --- | --- |
| *Predictors* | *Est.* | *CI* | *P* | *Est.* | *CI* | *P* |
| Control | 531.61 | 323.39 – 739.83 | **<0.001** | 0.16 | -0.04 – 0.36 | 0.121 |
| Control: T060 | 100.39 | -149.75 – 350.53 | 0.428 | 0.05 | -0.07 – 0.17 | 0.392 |
| Control: T120 | 88.39 | -161.75 – 338.53 | 0.485 | 0.04 | -0.08 – 0.16 | 0.507 |
| Control: T180 | 248.89 | -6.38 – 504.16 | 0.056 | 0.03 | -0.09 – 0.15 | 0.675 |
| LPS-1 | -68.97 | -363.25 – 225.31 | 0.643 | -0.08 | -0.36 – 0.21 | 0.590 |
| LPS-1: T060 | 84.97 | -268.62 – 438.56 | 0.635 | 0.02 | -0.14 – 0.19 | 0.786 |
| LPS-1: T120 | 322.97 | -30.62 – 676.56 | 0.073 | 0.15 | -0.02 – 0.32 | 0.074 |
| LPS-1: T180 | 480.47 | 123.24 – 837.71 | **0.009** | 0.25 | 0.09 – 0.42 | **0.003** |
| LPS-2 | 124.39 | -163.34 – 412.12 | 0.393 | 0.02 | -0.26 – 0.30 | 0.890 |
| LPS-2: T060 | -256.39 | -604.55 – 91.77 | 0.147 | 0.02 | -0.14 – 0.19 | 0.768 |
| LPS-2: T120 | -264.39 | -612.55 – 83.77 | 0.135 | 0.03 | -0.14 – 0.19 | 0.732 |
| LPS-2: T180 | -460.89 | -812.75 – -109.03 | **0.011** | 0.05 | -0.11 – 0.22 | 0.517 |
| *Abbreviations: LPS, lipopolysaccharide; LPS-1, LPS with support initiated at MAP <80mmHg; LPS-2, LPS with support initiated at MAP <65mmHg; Est., estimate; CI, confidence interval* | | | | | | |

**Table 4:** Linear mixed model outputs for mitoVO_2_

|  | Epidermal mitoVO_2_ | | | Intestinal serosa mitoVO_2_ | | | Liver mitoVO_2_ | | | Renal cortex mitoVO_2_ | | |
| --- | --- | --- | --- | --- | --- | --- | --- | --- | --- | --- | --- | --- |
| *Predictors* | *Est.* | *CI* | *P* | *Est.* | *CI* | *P* | *Est.* | *CI* | *P* | *Est.* | *CI* | *P* |
| Control | 14.60 | 10.47 – 18.74 | **<0.001** | 20.83 | 17.03 – 24.63 | **<0.001** | 17.91 | 14.71 – 21.11 | **<0.001** | 32.04 | 28.50 – 35.57 | **<0.001** |
| Control: T060 | -1.14 | -4.65 – 2.36 | 0.521 | 0.73 | -3.72 – 5.17 | 0.748 | 3.44 | -0.54 – 7.42 | 0.090 | -1.82 | -5.75 – 2.12 | 0.364 |
| Control: T120 | 0.94 | -2.57 – 4.44 | 0.599 | 0.55 | -3.85 – 4.95 | 0.806 | 1.05 | -2.93 – 5.03 | 0.604 | -2.96 | -6.93 – 1.01 | 0.143 |
| Control: T180 | 1.45 | -2.09 – 4.99 | 0.421 | 1.83 | -2.71 – 6.36 | 0.429 | 5.01 | 1.03 – 9.00 | **0.014** | -3.43 | -7.40 – 0.54 | 0.090 |
| LPS-1 | 2.33 | -3.56 – 8.22 | 0.436 | 0.03 | -5.37 – 5.44 | 0.990 | 0.06 | -4.47 – 4.58 | 0.980 | 1.62 | -3.36 – 6.59 | 0.523 |
| LPS-1: T060 | -0.84 | -5.84 – 4.16 | 0.742 | 0.22 | -6.07 – 6.50 | 0.946 | -2.43 | -8.09 – 3.22 | 0.398 | 5.19 | -0.38 – 10.75 | 0.068 |
| LPS-1: T120 | -7.12 | -12.17 – -2.06 | **0.006** | 1.74 | -4.61 – 8.10 | 0.589 | -0.46 | -6.14 – 5.23 | 0.874 | 4.29 | -1.30 – 9.88 | 0.132 |
| LPS-1: T180 | -6.50 | -11.53 – -1.47 | **0.012** | -2.73 | -9.05 – 3.59 | 0.396 | -5.99 | -11.77 – -0.20 | **0.043** | 0.15 | -5.44 – 5.74 | 0.958 |
| LPS-2 | 1.75 | -4.12 – 7.62 | 0.559 | 0.25 | -5.16 – 5.65 | 0.929 | 1.89 | -2.67 – 6.45 | 0.416 | 2.37 | -2.63 – 7.38 | 0.351 |
| LPS-2: T060 | -1.71 | -6.67 – 3.25 | 0.498 | -3.88 | -10.17 – 2.40 | 0.225 | -4.76 | -10.44 – 0.92 | 0.100 | 7.58 | 2.02 – 13.14 | **0.008** |
| LPS-2: T120 | -1.85 | -6.85 – 3.16 | 0.468 | -4.60 | -10.85 – 1.66 | 0.149 | -6.07 | -11.78 – -0.36 | **0.037** | 2.82 | -2.77 – 8.41 | 0.321 |
| LPS-2: T180 | 0.58 | -4.45 – 5.61 | 0.820 | -3.95 | -10.33 – 2.43 | 0.225 | -7.11 | -12.85 – -1.37 | **0.015** | -1.34 | -6.95 – 4.28 | 0.639 |
| *Abbreviations: mitoVO_2_, mitochondrial oxygen consumption; LPS, lipopolysaccharide; LPS-1, LPS with support initiated at MAP <80mmHg; LPS-2, LPS with support initiated at MAP <65mmHg; Est., estimate; CI, confidence interval* | | | | | | | | | | | | |

**Table 5:** Linear mixed model outputs for mitoPO_2_

|  | Epidermal mitoPO_2_ | | | Intestinal serosa mitoPO_2_ | | | Liver mitoPO_2_ | | | Renal cortex mitoPO_2_ | | | |  |
| --- | --- | --- | --- | --- | --- | --- | --- | --- | --- | --- | --- | --- | --- | --- |
| *Predictors* | *Est.* | *CI* | *P* | *Est.* | *CI* | *P* | *Est.* | *CI* | *P* | | *Est.* | *CI* | *P* | |
| Control | 67.39 | 52.80 – 81.97 | **<0.001** | 46.58 | 36.16 – 57.00 | **<0.001** | 70.06 | 60.34 – 79.78 | **<0.001** | | 69.01 | 60.60 – 77.43 | **<0.001** | |
| Control: T060 | -7.57 | -20.48 – 5.34 | 0.249 | 7.88 | -3.75 – 19.51 | 0.184 | 5.42 | -3.84 – 14.68 | 0.251 | | 1.02 | -6.63 – 8.68 | 0.793 | |
| Control: T120 | -3.76 | -16.66 – 9.15 | 0.567 | 17.68 | 6.05 – 29.31 | **0.003** | 5.73 | -3.54 – 14.99 | 0.225 | | -4.36 | -12.01 – 3.30 | 0.264 | |
| Control: T180 | -1.70 | -14.61 – 11.21 | 0.796 | 5.11 | -6.63 – 16.85 | 0.392 | 5.87 | -3.48 – 15.22 | 0.217 | | 3.17 | -4.48 – 10.82 | 0.416 | |
| LPS-1 | -1.06 | -21.61 – 19.50 | 0.920 | 6.74 | -8.07 – 21.55 | 0.371 | 3.34 | -10.41 – 17.09 | 0.633 | | -1.32 | -13.22 – 10.58 | 0.827 | |
| LPS-1: T060 | -4.35 | -22.60 – 13.91 | 0.640 | -3.90 | -20.43 – 12.62 | 0.643 | -5.15 | -18.31 – 8.01 | 0.442 | | 14.32 | 3.50 – 25.15 | **0.010** | |
| LPS-1: T120 | -18.17 | -36.42 – 0.09 | 0.051 | -17.20 | -33.80 – -0.60 | **0.042** | -9.70 | -22.80 – 3.40 | 0.146 | | 24.55 | 13.72 – 35.37 | **<0.001** | |
| LPS-1: T180 | -13.22 | -31.39 – 4.95 | 0.153 | -8.96 | -25.56 – 7.64 | 0.289 | -1.99 | -15.38 – 11.39 | 0.770 | | 8.38 | -2.45 – 19.20 | 0.129 | |
| LPS-2 | 10.55 | -10.08 – 31.18 | 0.315 | 5.36 | -9.37 – 20.09 | 0.474 | 1.99 | -11.76 – 15.74 | 0.776 | | 5.77 | -6.13 – 17.67 | 0.341 | |
| LPS-2: T060 | -3.30 | -21.55 – 14.95 | 0.722 | -3.76 | -20.21 – 12.69 | 0.653 | -9.74 | -22.84 – 3.36 | 0.145 | | 20.46 | 9.64 – 31.29 | **<0.001** | |
| LPS-2: T120 | -10.03 | -28.28 – 8.22 | 0.281 | -22.71 | -39.16 – -6.26 | **0.007** | -16.84 | -30.00 – -3.68 | **0.012** | | 17.09 | 6.26 – 27.91 | **0.002** | |
| LPS-2: T180 | -12.52 | -30.77 – 5.73 | 0.178 | -10.82 | -27.42 – 5.78 | 0.201 | -6.45 | -19.61 – 6.71 | 0.336 | | 0.52 | -10.31 – 11.34 | 0.925 | |
| *Abbreviations: mitoPO_2_, mitochondrial oxygenation; LPS, lipopolysaccharide; LPS-1, LPS with support initiated at MAP <80mmHg; LPS-2, LPS with support initiated at MAP <65mmHg; Est., estimate; CI, confidence interval* | | | | | | | | | | | | | |  |

**Table 6:** Linear mixed model outputs for sublingual microcirculation; microvascular perfusion parameters

|  | Total vessel density | | | Functional capillary density | | | Proportion of perfused vessel | | |
| --- | --- | --- | --- | --- | --- | --- | --- | --- | --- |
| *Predictors* | *Est.* | *CI* | *P* | *Est.* | *CI* | *P* | *Est.* | *CI* | *P* |
| Control | 25.04 | 23.24 – 26.83 | **<0.001** | 22.69 | 20.97 – 24.42 | **<0.001** | 0.89 | 0.86 – 0.91 | **<0.001** |
| Control: T060 | 0.65 | -1.36 – 2.66 | 0.524 | 1.21 | -0.69 – 3.11 | 0.210 | 0.05 | 0.01 – 0.08 | **0.005** |
| Control: T120 | 0.97 | -1.04 – 2.98 | 0.342 | 1.67 | -0.23 – 3.57 | 0.084 | 0.05 | 0.02 – 0.08 | **0.003** |
| Control: T180 | 1.14 | -0.88 – 3.15 | 0.265 | 1.48 | -0.41 – 3.38 | 0.124 | 0.04 | 0.01 – 0.07 | **0.019** |
| LPS-1 | -0.14 | -2.56 – 2.29 | 0.910 | 0.03 | -2.30 – 2.36 | 0.980 | 0.01 | -0.02 – 0.04 | 0.552 |
| LPS-1: T060 | -0.03 | -2.75 – 2.70 | 0.985 | -0.91 | -3.48 – 1.67 | 0.485 | -0.04 | -0.09 – 0.00 | 0.065 |
| LPS-1: T120 | -1.10 | -3.83 – 1.63 | 0.426 | -1.83 | -4.40 – 0.75 | 0.162 | -0.03 | -0.08 – 0.01 | 0.119 |
| LPS-1: T180 | -2.72 | -5.48 – 0.05 | 0.055 | -3.23 | -5.85 – -0.62 | **0.016** | -0.04 | -0.08 – 0.01 | 0.102 |
| LPS-2 | 2.12 | -0.43 – 4.66 | 0.102 | 2.10 | -0.34 – 4.54 | 0.091 | 0.03 | -0.01 – 0.06 | 0.105 |
| LPS-2: T060 | -0.50 | -3.38 – 2.38 | 0.731 | -1.15 | -3.87 – 1.56 | 0.401 | -0.05 | -0.10 – -0.00 | **0.035** |
| LPS-2: T120 | -2.85 | -5.70 – -0.01 | **0.050** | -4.15 | -6.84 – -1.47 | **0.003** | -0.08 | -0.13 – -0.04 | **0.001** |
| LPS-2: T180 | -2.28 | -5.19 – 0.62 | 0.121 | -3.01 | -5.75 – -0.27 | **0.032** | -0.06 | -0.11 – -0.01 | **0.012** |
| *Abbreviations: LPS, lipopolysaccharide; LPS-1, LPS with support initiated at MAP <80mmHg; LPS-2, LPS with support initiated at MAP <65mmHg; Est., estimate; CI, confidence interval* | | | | | | | | | |

**Table 7:** Linear mixed model outputs for sublingual microcirculation; red blood cell dynamics

|  | Red Blood Cell Velocity | | | Tissue Red Blood Cell Perfusion | | |
| --- | --- | --- | --- | --- | --- | --- |
| *Predictors* | *Est.* | *CI* | *P* | *Est.* | *CI* | *P* |
| Control | 274.78 | 259.34 – 290.22 | **<0.001** | 61.73 | 54.28 – 69.18 | **<0.001** |
| Control: T060 | 37.79 | 17.29 – 58.29 | **<0.001** | 8.17 | 0.30 – 16.05 | **0.042** |
| Control: T120 | 33.43 | 12.93 – 53.93 | **0.002** | 10.65 | 2.77 – 18.52 | **0.009** |
| Control: T180 | 20.44 | -0.06 – 40.94 | 0.051 | 6.71 | -1.16 – 14.59 | 0.094 |
| LPS-1 | 12.34 | -8.39 – 33.06 | 0.240 | 2.45 | -7.62 – 12.53 | 0.630 |
| LPS-1: T060 | -56.44 | -84.27 – -28.61 | **<0.001** | -11.02 | -21.68 – -0.35 | **0.043** |
| LPS-1: T120 | -44.35 | -72.19 – -16.52 | **0.002** | -14.02 | -24.69 – -3.35 | **0.011** |
| LPS-1: T180 | -40.52 | -68.72 – -12.31 | **0.005** | -18.16 | -29.00 – -7.33 | **0.001** |
| LPS-2 | 19.02 | -2.82 – 40.87 | 0.087 | 10.28 | -0.27 – 20.82 | 0.056 |
| LPS-2: T060 | -63.05 | -92.43 – -33.67 | **<0.001** | -11.71 | -22.97 – -0.45 | **0.042** |
| LPS-2: T120 | -76.50 | -105.49 – -47.51 | **<0.001** | -25.52 | -36.67 – -14.37 | **<0.001** |
| LPS-2: T180 | -55.91 | -85.36 – -26.47 | **<0.001** | -18.00 | -29.36 – -6.64 | **0.002** |
| *Abbreviations: LPS, lipopolysaccharide; LPS-1, LPS with support initiated at MAP <80mmHg; LPS-2, LPS with support initiated at MAP <65mmHg; Est., estimate; CI, confidence interval* | | | | | | |
